# Unraveling the mystery of alien hand syndrome: when your hand has a mind of its own

**DOI:** 10.1186/s13023-025-04000-y

**Published:** 2025-10-06

**Authors:** Khaled Moghib, Trisha Shivashankar, Thoria I. Essa Ghanm, Mona I. Elshamy, Eman G. Allam, Salomon Izere, Md. Al Hasan Mia, Muhannad Wael Abu Arafeh, Mostafa Meshref

**Affiliations:** 1 Faculty of Medicine, Cairo University, Cairo, Egypt; 2 Medical Research Group of Egypt, Negida Academy, Arlington, MA USA; 3 Bharati Vidyapeeth Medical College, Pune, India; 4 Faculty of Medicine, Mansoura University, Mansoura, Egypt; 5 Faculty of Medicine, Ain Shams University, Cairo, Egypt; 6 Dhaka Medical College, Dhaka, Bangladesh; 7 University of Rwanda College of Medicine and Health Sciences, Kigali, Rwanda; 8 Faculty of Medicine, Al-Azhar University, Cairo, Egypt; 9 Hadassah Medical Center, Jerusalem, Israel; 10 Department of Neurology, Faculty of Medicine, Al-Azhar University, Cairo, Egypt

**Keywords:** Alien hand syndrome, Stroke, Hemiparesis, Corpus callosum, Neurodegeneration

## Abstract

**Background:**

Alien Hand Syndrome (AHS) is a rare neurological disorder characterized by involuntary, complex movements of a limb, often with a sense of estrangement from the affected hand. Initially described in 1908, AHS has since been associated with various neurological conditions, including strokes, neurodegenerative diseases, tumors, and surgical interventions affecting the corpus callosum and frontal lobes.

**Objective:**

This study aims to provide a comprehensive review of etiology, clinical manifestations, neuroanatomical basis, and differential diagnosis of AHS, synthesizing findings from published case reports and literature.

**Methods:**

This scoping review followed PRISMA-ScR guidelines to systematically analyze AHS case reports from PubMed (2010–2025). Two independent reviewers screened studies using predefined criteria, extracting demographic, clinical, neuroimaging, and treatment data. Eligible case reports required a confirmed AHS diagnosis with complete clinical and neuroimaging documentation. Data synthesis combined descriptive statistics and qualitative analysis to map AHS characteristics across subtypes.

**Results:**

A total of 72 cases were reviewed, with a mean patient age of 59.58 years (SD 18.24), ranging from 9 to 89 years. Males accounted for 48.6% (35 cases), while females represented 51.4% (37 cases). The most frequently implicated brain regions were the corpus callosum, supplementary motor area, and posterior parietal cortex. AHS was commonly associated with stroke, neurodegenerative diseases (e.g., corticobasal syndrome, Creutzfeldt-Jakob disease), and brain tumors. The disorder was categorized into three subtypes, each with distinct clinical presentations and underlying neuropathology. Differential diagnoses included psychiatric disorders, movement disorders, and body schema disturbances.

**Conclusion:**

AHS remains a rare and complex neurological disorder with diverse etiologies and clinical manifestations. Accurate diagnosis requires thorough clinical evaluation and neuroimaging to differentiate AHS from psychiatric and other neurological conditions. Further research is needed to elucidate its pathophysiology and develop targeted therapeutic approaches.

## Background

In 1908, Goldstein described a 57-year-old woman with alien hand syndrome whose left hand acted independently, once grabbing her throat and requiring great effort to restrain. An autopsy revealed corpus callosum and right hemisphere strokes [1]. Akelaitis described another patient whose left hand would uncontrollably do the opposite of her right hand following a corpus callosum section after a few years. Akelaitis named this syndrome diagnostic dyspraxia [2]. In 1972, Brion and Jedynak coined the term “alien hand” to characterize midline brain tumor patients who denied ownership of one hand [3]. Although the ‘alien’ hand appears to move independently, patients are aware of its movements but have no control over them. However, “alien hand” describes the patient’s emotional dissociation from the limb [1, 2, 4, 5].

AHS can result from neurodegenerative illnesses, including corticobasal syndrome (CBS), Lewy body dementia (LBD), Creutzfeldt-Jakob disease (CJD), and subacute midline tumors. AHS can happen suddenly after an aneurysm, stroke, or surgery on the corpus callosum [6]. It is also linked to damage in the thalamus, cingulate gyrus, supplementary motor area, posterior parietal cortex, and corpus callosum [7, 8].

After analyzing the literature, frontal and callosal alien hands were identified. The former is based on the corpus callosum. The anterior cerebral artery or neighboring arteries can be damaged by ischemia or rupture, or the anterior callosum can be surgically removed to treat epileptic episodes that do not respond to medicine [9]. The condition predominantly affects the frontal lobes, which control executive processes and voluntary movements. Disconnection syndrome is one of its symptoms. Damage to the hemispheres disrupts their harmonic communication, causing one hand to move automatically while the other functions voluntarily [10]. Mental illness differential diagnosis. It may be hard to distinguish AHS symptoms from mental disorders [11].

## Methodology

### Study design objective

This scoping review was conducted following the PRISMA-ScR [12] (Preferred Reporting Items for Systematic Reviews and Meta-Analyses extension for Scoping Reviews) guidelines to systematically map the existing literature on Alien Hand Syndrome (AHS). The objective was to identify and synthesize case reports on AHS, focusing on demographic trends, clinical manifestations, neuroanatomical correlates, and treatment approaches.

### Databases searched and search strategy

A comprehensive search strategy was employed in PubMed, covering case reports published between 2010 and 2025. The search terms included MeSH keywords and Boolean operators to capture all relevant studies: (“Alien Hand Syndrome” or “Anarchic Hand Syndrome” or “Alien Limb Syndrome” or “Dr. Strangelove Syndrome”) and (“Pathophysiology” or “Etiology” or “Neuroimaging” OR “Treatment” or “Differential Diagnosis”) and (“Corpus Callosum” or “Frontal Lobe” or “Parietal Lobe” or “Supplementary Motor Area”).

### Eligibility criteria

The eligibility criteria were structured to include peer-reviewed case reports with detailed demographic (age, sex) and clinical data, confirmed AHS diagnosis, and neuroimaging findings. Exclusion criteria removed studies with incomplete data, duplicates, or non-case report or case series publications (e.g., reviews, editorials), and non-English articles.

Data extraction was performed using a standardized form, capturing: Demographics (age, sex, handedness), Clinical features (AHS subtype, key symptoms), Neuroimaging findings (lesion location, imaging modality), Treatment approaches, and outcomes.

The extracted data were synthesized through descriptive statistics (mean age, sex distribution) and qualitative narrative analysis, categorizing cases by AHS subtype (callosal, frontal, posterior) and lesion localization. Results were presented in tables and summarized to highlight trends in pathophysiology and management. If the paper included more than one case, we extracted each case sparsely in tables.

### Study selection

**Fig. 1:**
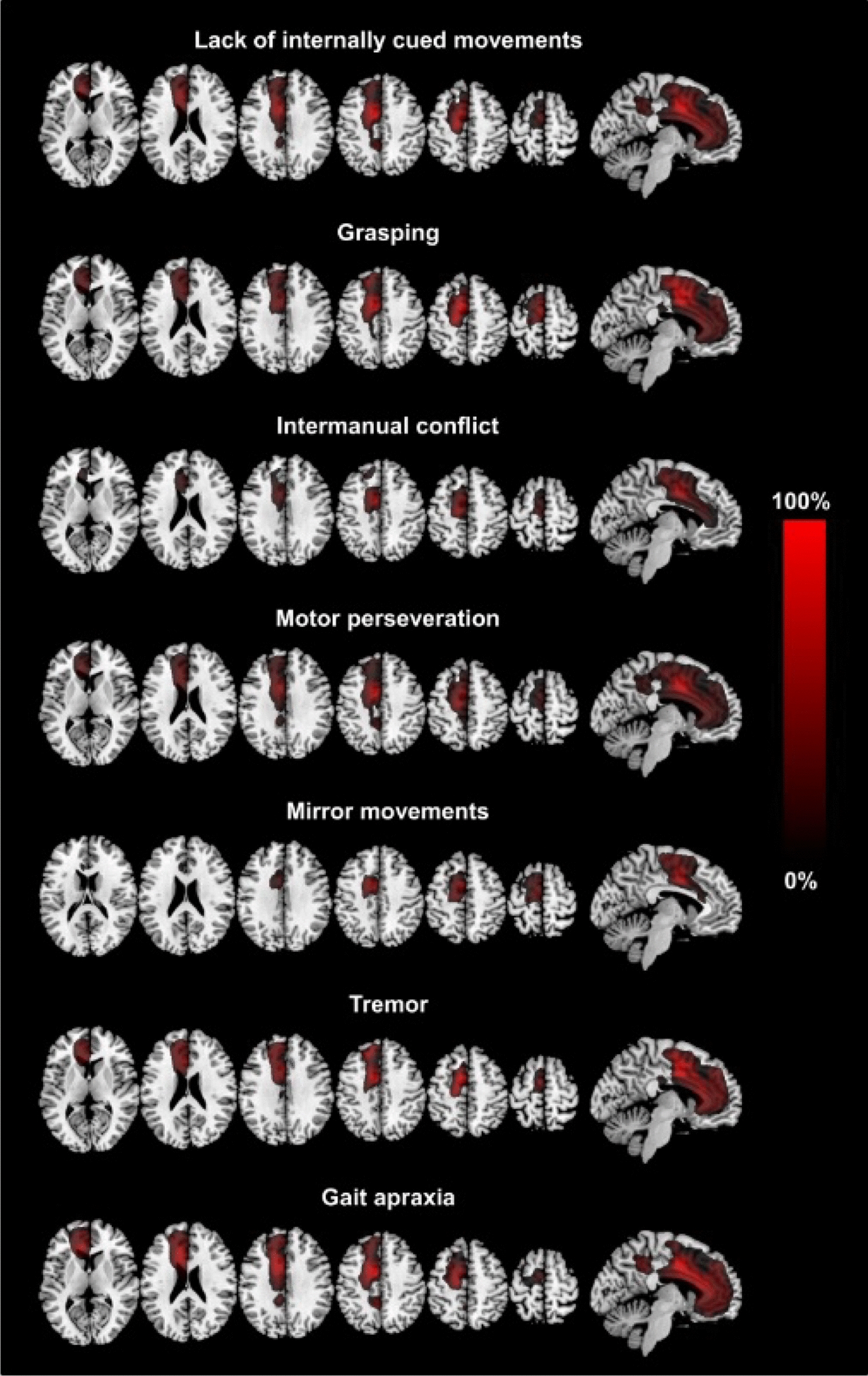
Summation lesion maps on T1-standard MRI showing voxels damaged in > 25% of patients for each clinical sign: frontal, callosal, and posterior variants [21]

**Fig. 2:**
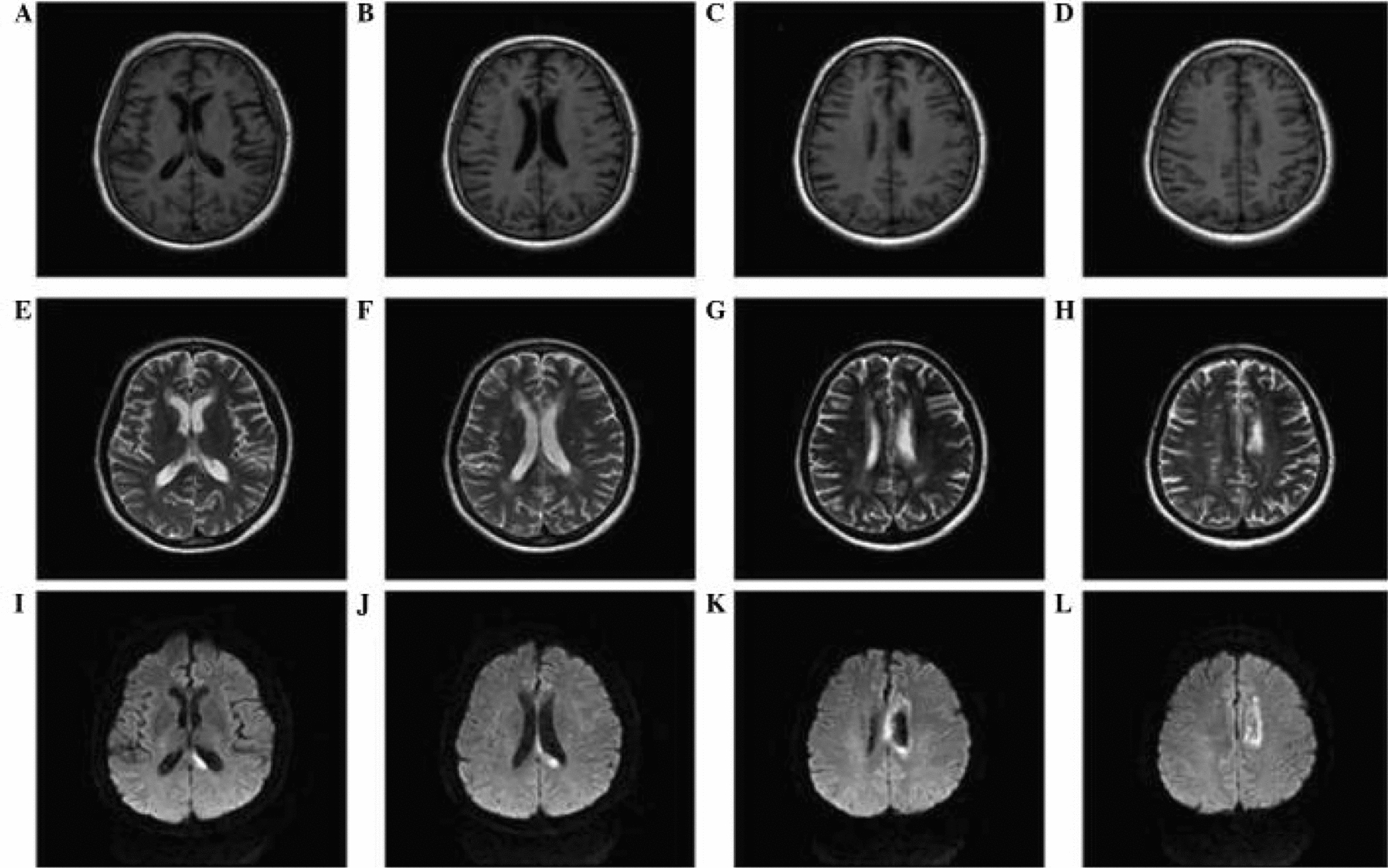
Multimodal MRI of the left corpus callosum: T1 (hypo-intensity in the splenium  (**A**–**B**) & body (**C**–**D**)), T2 (hyper-intensity in the splenium  (**E**–**F)** & body (**G**–**H**)), and diffusion-weighted (high signal intensity in the splenium (**I**–**J**) & body (**K**–**L**)) images confirming infarction [15]

**Fig. 3:**
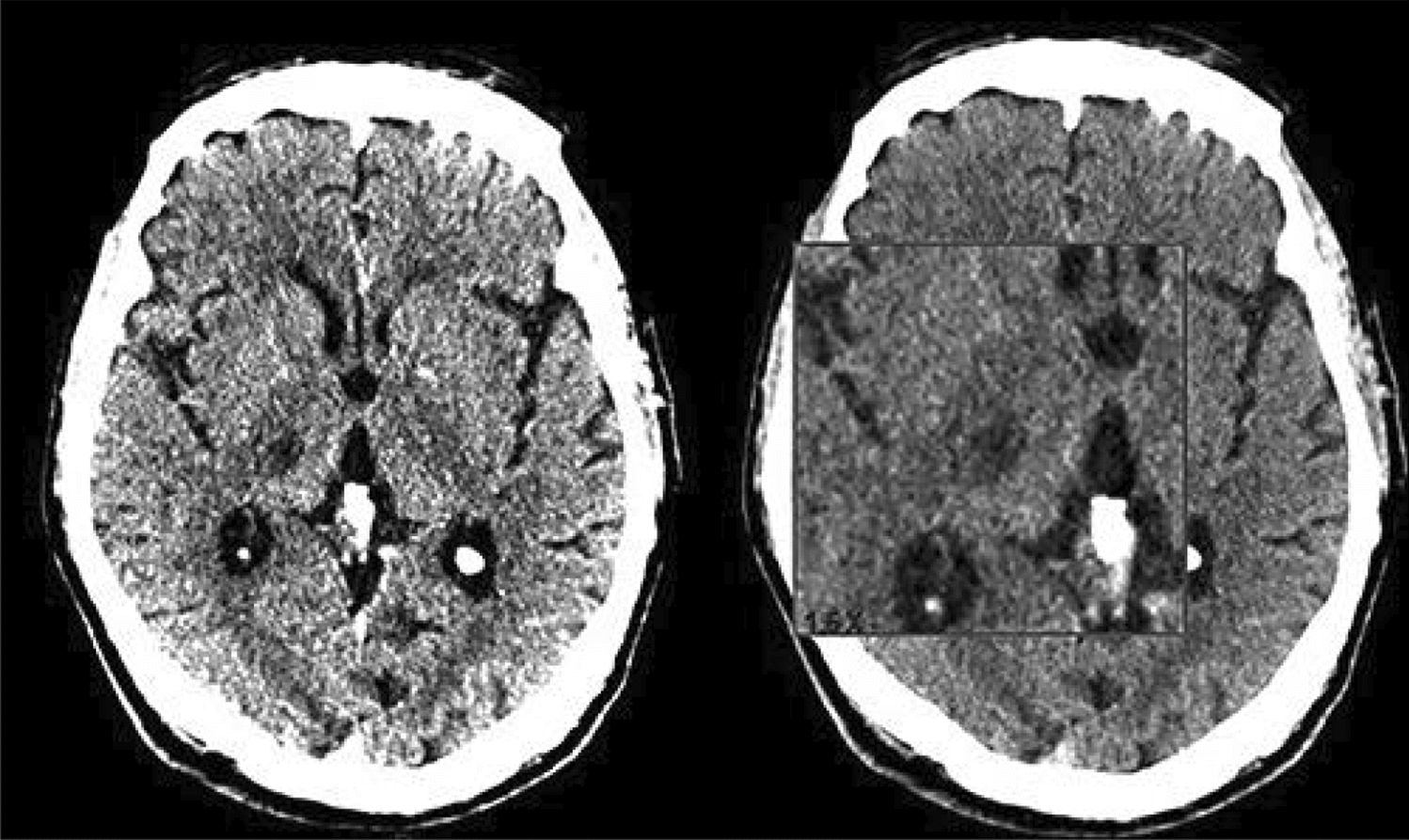
24-h computed tomography brain scan showing a new-onset right thalamic ischemic lesion [55]

Two independent researchers conducted a dual-phase screening process. Initially, titles and abstracts were reviewed for relevance. Subsequently, full-text articles were evaluated against the inclusion criteria. Any disagreements between reviewers were resolved through discussion or consultation with a third reviewer, ensuring rigorous and unbiased selection (Figs. 1, 2 and 3).

## Results

### Data search and screening

Our Search identified 118 studies from one database (PubMed 2010–2025), with no duplicates removed, and 118 studies were eligible for screening. After further rigorous screening, a final 62 studies were included, with 72 cases.

### Characteristics of included studies

A total of 72 cases were included in this review. The mean age of the cases was 59.58 years (standard deviation: 18.24 years), with ages ranging from 9 to 89 years. The majority of cases were male (35 cases, 48.6%), while females accounted for 37 cases (51.4%). This demographic distribution highlights the broad age range and slight male predominance in the reviewed case reports. The inclusion of diverse cases provides a comprehensive understanding of the conditions across various age groups and sexes. (Table 1).

**Table 1:** Literature review report

ID	Sex	Age	Unilateral/bilateral	Alien hand type	Clinical manifestations	Imaging/neuroanatomy	Duration of symptoms	Response to stimuli or situations	Accompanied disease	Treatment	Damaged cerebral area
McLean et al. [4]	Male	39	Unilateral (Rt. hand)	callosal type	Involuntary movements of the right hand (tapping on the bed), intermanual conflict, and paresis of the right leg. When attempting to drink, the left hand pushed the cup away	Centralized ischemic lesion in the trunk of the corpus callosum due to localized damage to the pericallosal pial plexus by the hematoma	2 weeks	Intermanual conflict during everyday tasks; he described the left hand as "evil" but recognized it as his own	Subarachnoid hemorrhage	The aneurysm was coiled	Corpus callosum and pericallosal pial plexus
Korsakofa et al., [77]	Female	25	Unilateral (Lt. hand)	Frontal type	Left posterior alien hand	NA	NA	NA	Subarachnoid hemorrhage	NA	NA
McLean et al. [4]	Male	39	Unilateral (Rt. handed)	Callosal type	Left hemiparesis, involuntary movement of the right hand, and intermanual conflict	MRI: Centralized ischemic lesion in the corpus callosum truncus due to damage to the pericallosal pial plexus by hematoma	6 months	Named his left hand "evil." The right hand attempted to drink, left hand pushed the cup away	Subarachnoid hemorrhage	Resolved spontaneously after aneurysm treatment	Corpus callosum
Rahwan et al. [42]	Male	70	Unilateral (Lt. hand)	posterior	Rapid, abrupt, uncontrollable movements of the left arm and leg, moving upwards when attempting to grasp objects	CT brain: Right MCA stroke. Angiography: Occlusion of the inferior branch of the M2 segment of the right MCA	1 week	Demonstrated uncontrollable movements when attempting intentional movements	stroke	Low-dose clonazepam (0.5 mg twice daily)	Temporoparietal and internal capsule region
Le et al. [44]	Male	88	Unilateral (Lt. hand)	posterior	Intermittent involuntary movement of the left forearm and hand (e.g., slapping himself while napping)	Brain MRI: Acute infarction in the right temporal lobe, right parietal cortex, and right parietal subcortex	1 day	Involuntarily slapping himself while napping	stroke	Empiric aspirin and statin therapy	Right temporal lobe, right parietal cortex, and right parietal subcortex
Mammi et al., [78]	Female	44	Unilateral (Lt. hand)	posterior	Sudden movement of the left side of her body caused a fall	Right temporo‑parietal damage	NA	NA	Stroke	NA	Right temporo‑parietal damage
Panikath et al., [79]	Female	77	Unilateral (Lt. hand)	posterior	Left hand flinging across her visual field; purposeful movements, loss of control for 30 min	CT and MRI: Acute infarcts in both parietal lobes	30 min	Attempts to control the left hand with the right hand were unsuccessful	Stroke	Anticoagulation	Both parietal lobes
Russo et al., [55]	Male	80	Unilateral (Lt. hand)	posterior	Self-inflicted trauma; paraesthesia on left side; spontaneous levitation of left arm	CT: Old malacic lesion in right occipitoparietal region; later scan: ischemic lesion in right thalamus	4 days	Asked for the arm to be tied to the bed	stroke	NA	Right thalamus
Nowak et al., [80]	Female	78	Unilateral (Lt. hand)	posterior	She complained of problems controlling her left arm and hand. The left arm and hand were “moving unintended”, “having a will of their own”. When washing or dressing, her left hand interfered with and disturbed the activities of her right hand. She denied loss of possession but described her left arm as “annoying”, “performing out of her will”, and “not obeying her commands”.	Computed tomography revealed an ischemic lesion within the territory of the right posterior cerebral artery. MRI showed hypodense lesions within the right posterior thalamus, right occipital, medial temporal, and inferior parietal lobes	9 days	Suppressed movements by placing the arm and hand under the blanket in bed, under a wheelchair table, or wedging it between her legs. Guided attention to the left visual field temporarily stopped movements for a few seconds. Movements did not stop when distracted (e.g., talking to the examiner or caregiver)	stroke	A 12-week rehabilitation program: daily physiotherapy, occupational therapy, and neuropsychological training	Right posterior thalamus, right occipital, medial temporal, and inferior parietal lobes
Zainudin et al., [81]	Female	57	Unilateral (Lt. hand)	Three types (frontal, callosal, posterior)	Frontal symptoms: grasping, groping, and difficulty releasing objects; callosal type: intermanual conflict; posterior type: arm levitation, mild hemiparesis, hemisensory loss	CT: right temporal infarct and acute corpus callosum infarct extending from right genu to left splenium. CTA: stenosis of M1 of MCA, left PCA, and hypoplastic right PCOM	9 months	Left-hand counteracts right-hand actions, unresponsive to verbal commands or visual cues (ideomotor apraxia)	stroke	Mirror box therapy, limb restraint, CBT, bimanual hand training, clonazepam	Corpus callosum (callosal disconnection syndrome)
Schaefer et al., [82]	Female	69	Unilateral (Rt. hand)	Callosal type	Unwanted right-hand jerks, an electrified feeling, and occasional attempts to slap her face. No intermanual conflict observed	Missing data for CT/MRI	NA	Involuntary movements predominated at night; controlled during the day	stroke,	NA	Left cerebral hemisphere
Rahwan et al. [42]	Male	70	Unique (both upper/lower left limbs)	Posterior type	Abrupt, involuntary movements in the left upper/lower limbs, especially during voluntary movement. The arm moved in different directions involuntarily before limb weakness	CT: well-established MCA stroke. Angiography: occlusion of the inferior branch of the M2 segment of the right MCA	1 month	Movements occurred throughout the day against his will	stroke	Low-dose clonazepam 0.5 mg BID; ceased 1 month post-discharge	Right temporal and internal capsule regions
Panikath et al., [79]	Female	77	Unilateral (Lt. hand)	Frontal type	Left hand stroked her face and hair involuntarily; she resolved after half an hour. On recovery, left hemiparesis	CT/MRI: Acute infarction in both parietal lobes	30 min	Attempted to control the left hand with the right hand, unsuccessful. Resolved spontaneously after 30 min	stroke	Resolved spontaneously, anticoagulant resumed	Both parietal lobes
Nowak et al. (1st case) [30]	Female	54	Unilateral (Rt. hand)	Frontal type	Rt. hemiparesis, inability to release grip, continuous groping, fumbling, impaired bimanual coordination. No apraxia, neglect, or writing impairment	CT: Hypodense lesion in left anterior corpus callosum, paramedian white matter, and cortex	28 weeks	Attempted to control involuntary behavior by clamping a hand under the thigh or wedging it between the legs	stroke	Rehabilitation therapy	Corpus callosum, paramedian cortex
Nowak et al. (2nd case) [30]	Female	71	Unilateral (Rt. handed)	Frontal type	Frequent impulsive reaching, grasping, and difficulty releasing objects. The right hand interfered with the left hand's purposeful actions. No neglect or writing impairment	MRI: Ischemic lesion in the anterior corpus callosum and overlying white matter	37 weeks	Suppressed the right hand with the left hand, but strategies didn’t persist long-term	stroke	Rehabilitation therapy	Corpus callosum
Kloesel et al. [83]	Female	70	Unilateral (Rt. handed)	Posterior type	Involuntary right arm movements, repetitive face tapping. Increased with emotional stress, no intermanual conflict or grasping behavior	MRI: Acute left parietal stroke	24 h	Involuntary movements increased with stress, resolved spontaneously within 24 h	stroke	Resolved spontaneously	Parietal lobe
BRU et al., [84]	Male	21	Unilateral (Rt. hand)	Frontal type	Unwittingly grabbed objects with his right hand, unable to release grip voluntarily; needed assistance from his left hand	Brain MRI: Hyperintense periventricular to cortico-subcortical changes; diffuse restrictive signal changes parafalcine left fronto-parietal region; ischemic signal changes (parietal and lateral ventricle regions)	19 weeks	Unable to release grip voluntarily; used verbal cues, distraction tasks, and visualization strategies	stroke	Rehabilitation therapy	Left fronto-parietal region
Bartolo et al., [46]	Female	61	Unilateral (Rt. hand)	Posterior type	Right hemiparesis, intermittent involuntary movements (slow onset); levitation of right arm; right hand stroked the bed, failed to recognize affected limb as her own, personified it, no intermanual conflict	CT scan: Infarct in left cortico-subcortical posterior cerebral artery territory; MRI: Ischemic lesions (thalamus, parahippocampal gyrus, inferior/posterior temporal lobe, splenium of corpus callosum, occipital cortex)	4 months	Did not seem aware of the problem; no attempts to intervene	stroke	Neurorehabilitation (8 weeks)	Posterior thalamus
Higgins et al. [58]	Female	81	Unilateral (Rt. hand)	Callosal type	Patient reported autonomous right-hand movements, dominantly touching her chest and face. She thought her hand was possessed. No additional symptoms	CT and CTA: No acute abnormalities; MRI: Significant restricted diffusion in the left parietal lobe anterior and superior to the Sylvian fissure, compatible with acute stroke	Acute onset	Went to ED	stroke	Rehabilitation therapy	Left parietal lobe
Yuan et al., [85]	Female	71	Unilateral (Lt. hand)	Callosal type	Involuntary movements, the claimed hand belonged to someone else; intermanual conflict during bimanual tasks	MRI: Corpus callosum infarction; MRA: Stenosis in bilateral middle and left posterior cerebral arteries	2 weeks	NA	corpus callosum infarction	Platelet aggregation inhibitor; rehabilitation	Corpus callosum
Qu et al., [86]	Female	57	Bilateral (right/left hands)	Mixed (callosal-frontal types)	Uncoordinated hand movements; involuntary grasp reflex (right hand, frontal variant); intermanual conflict (left hand, callosal variant)	MRI: ischemic strokes in the corpus callosum and left frontal lobe. MRA: stenosis of the A4 segment of the ACA, poor visualization of the left MCA. Edema in the corpus callosum	3 months	Involuntary movements were coordinated during purposeful left-hand use	corpus callosum infarction	Rehabilitation via verbal-cue therapy	Frontal lobe, corpus callosum
Mahawish et al., [87]	Female	58	Unilateral (Lt. hand)	Callosal type	Intermanual conflict (e.g., opening cupboards with right hand, closing with left hand)	MRI: Corpus callosum infarction in the left hemisphere	2 months	NA	corpus callosum infarction	NA	Left corpus callosum
Ma et al., 1st case [88]	Male	69	Unilateral (Lt. hand)	Callosal type	Intermanual conflict symptoms on admission	Right genu and splenium of the corpus callosum, right cingulate gyrus: Hyperintensity on T2 and DWI, hypointense on T1 and ADC	5 days	Intermanual conflict symptoms on admission	corpus callosum infarction	Antiplatelet and open collateral circulation	Right corpus callosum and cingulate gyrus
Ma et al. 2nd case [88]	Female	63	Unilateral (Rt. hand)	Callosal type	Intermanual conflict symptoms after admission	Left genu of the callosum and left cingulate gyrus: Hyperintensity on T2 and DWI, hypointense on T1 and ADC	10 days	Intermanual conflict symptoms after admission	corpus callosum infarction	Antiplatelet and open collateral circulation	Left corpus callosum and cingulate gyrus
Ma et al. 3rd case [88]	Female	52	Unilateral (Rt. hand)	Callosal type	Intermanual conflict symptoms after admission	Left anterior cingulate gyrus and left genu of corpus callosum: Hyperintensity on T2 and DWI, hypointense on T1 and ADC	5 days	Intermanual conflict symptoms after admission	corpus callosum infarction	Antiplatelet and open collateral circulation	Left corpus callosum and cingulate gyrus
Ma et al., 4th case [88]	Male	47	Unilateral (Lt. hand)	Callosal type	Intermanual conflict symptoms after admission	Multiple cerebral infarctions involving the right cingulate gyrus and the corpus callosum at the genu: Hyperintensity on DWI	4 days	Intermanual conflict symptoms after admission	corpus callosum infarction	Antiplatelet and open collateral circulation	Right corpus callosum and cingulate gyrus
Ma et al. 5th case [88]	Female	61	Unilateral (Rt. hand)	Callosal type	Strong grip symptoms in the affected hand	DWI: New lesions in the left frontal lobe, left genu and splenium of the corpus callosum, left cingulate gyrus	12 days	Strong grip symptoms in the affected hand	corpus callosum infarction	Antiplatelet and open collateral circulation	Left corpus callosum and cingulate gyrus
Basu et al., [89]	Female	15	Unilateral (Lt. hand)	Callosal type	Difficulty releasing objects, left hand acting in opposition to right hand actions, e.g., closing doors when attempting to open them, interfering with purposeful activities. No arm elevation or self-injurious behavior was noted	Marked callosal atrophy with loss of direct interhemispheric connections except for parts of the genu and splenium	20 months	Managed episodes by physically moving away from the situation producing the conflict	callosal lesion	NA	Marked callosal atrophy
Ma et al., [90]	Male	57	Unilateral (Lt. hand)	Callosal type	Loss of bimanual coordination: the left hand performed opposite movements to the right hand. Complained that his left hand did not belong to him but to someone else	Brain MRI showed low signal intensity in the genu, body, and splenium of the right corpus callosum on T1-weighted images, with high signal intensity in the same regions on DWI, T2-weighted, and FLAIR images	2 weeks	NA	callosal lesion	Symptomatic and supportive treatment	Genu, body, and splenium of the right corpus callosum
Jang et al., [91]	Female	72	Unilateral (Rt. hand)	Callosal type	At 4 weeks post-onset, the patient displayed autonomous complex movements of the right hand against her will: (1) impulsively reaching and grasping objects, (2) compulsively manipulating objects (e.g., putting food in her mouth even if her mouth was already full), and (3) difficulty voluntarily releasing objects. No intermanual conflict or denial of ownership observed	MRI (16 days post-onset) showed infarct lesions confined to the anterior left cingulate gyrus and corpus callosum (genu to anterior splenium)	4 weeks	NA	callosal lesion	NA	Left cingulate gyrus and corpus callosum (genu to anterior splenium)
Krausse et al.,[92]	Male	79	Unilateral (Lt. hand)	Callosal type	Involuntary grasping, intermanual conflict; no sense of ownership of the left hand	MRI: Complete agenesis of corpus callosum, colpocephaly; no spinal cord pathology	1 year	NA	callosal lesion	Ropinirole (1.5 mg)	Agenesis of corpus callosum
Faber et al. [62]	Male	56	Unilateral (Rt. hand)	Callosal type	Involuntary right-hand movements (e.g., slapping forehead, interfering with left hand’s actions), impaired bimanual coordination. Wife noted movements reduced with humor	MRI: Lesion in left paracallosal region (genu to splenium of corpus callosum); infarct affecting left pericallosal artery	NA	Tried to restrain the right hand using the left hand; movements reduced when the wife joked around	callosal lesion	NA	Left pericallosal region
Erdal et al. [57]	Male	42	Unilateral (Rt. hand)	posterior	Sudden involuntary movements of the right arm after visual loss in the right visual hemifield. The arm was described as acting like a puppet’s arm during a migraine attack	MRI: Normal	1 week	The arm acted involuntarily, e.g., changing car gears involuntarily	Migraine	Simple analgesic treatment	NA
Erdal et al. [57]	Male	42	Unilateral (Rt. hand)	Post. type	Involuntary right arm movements with visual loss in the right visualhemifielde the right arm felt like it belonged to someone else	MRI and MRA: Normal findings	10–15 min	Unable to control the hand; just watched it	Migraine	Resolved spontaneously	Occipital lobe
Gallant et al., [37]	Male	41	Unilateral (Lt. hand)	posterior	Hypoesthesia, involuntary movements (e.g., grabbing doorknobs), and the need for restraint with the right hand	MRI: White matter changes interpreted as edema or gliosis; no radiation necrosis	4 months	Episodes startled the patient; required right-hand restraint	Neoplastic disease	NA	Right parietal lobe
Perren et al., [93]	Female	55	Unilateral (Lt. hand)	posterior	Left-sided hemiataxia-hemiparesis, left hemisensory loss, and short-lasting episodes of an alien left hand due to lesions of the internal capsule and right thalamus, extending into the mesencephalon. Associated with extensive edema	MRI showed gadolinium-enhancing lesions in the right thalamus, internal capsule, caudate nucleus, and mesencephalon with perifocal edema. Stereotactic brain biopsy showed a sclerosing inflammatory process with demyelination, macrophage infiltration, and astrocyte hypertrophy	NA	NA	Neoplastic disease	Pulsed oral dexamethasone (12 mg, 5 days/month) and daily mycophenolate mofetil (2 × 1 g/day)	Right thalamus, internal capsule, caudate nucleus, mesencephalon
Ciaralito et al., [94]	Male	56	Unilateral (Lt. hand)	posterior	Exhibited levitation of the left arm	MRI revealed diffusion restriction predominantly in the right parietal cortex, caudate, and putamen	2 months	NA	Creutzfeldt–Jakob Disease	NA	Right parietal cortex
Barnwall et al., [95]	Male	57	Unilateral (Rt. hand)	posterior	Complained of classical alien limb phenomenon with involuntary motor activity of the right upper limb	MRI showed extensive cortical diffusion restriction mainly affecting the left frontal lobe and cingulate gyrus (DWI). FLAIR signal of the cortex in the left frontotemporal-parietal lobes without pathological contrast enhancement	NA	NA	Creutzfeldt–Jakob Disease	Levetiracetam	Temporoparietal lobe
Martinez et al., [96]	Female	82	Unilateral (Lt. hand)	posterior	Left posterior alien hand	Diffusion-weighted brain MRI showed hyperintensities in the right cerebral cortex and basal ganglia. Cranial CT did not show abnormalities	2 weeks	NA	Creutzfeldt–Jakob Disease	NA	The right cerebral cortex and basal ganglia
Zannino et al., [97]	Female	66	Unilateral (Lt. hand)	posterior	Objects fell from her left hand without her awareness. Developed spontaneous, involuntary tremor-like movements of the same hand	MRI DWI documented restricted diffusion in the striatum bilaterally (more on the right) and in the right thalamic pulvinar nuclei. The same areas appeared hyperintense on FLAIR sequences	Recently	NA	Creutzfeldt–Jakob disease	NA	Striatum bilaterally and the right thalamus
Porcel et al., [98]	Male	60	Unilateral (Rt. hand)	posterior	He exhibited picking movements with his right hand, which he did not perceive as alien	Brain MRI showed only minimal atrophy of the left posterior frontal and anterotemporal lobes	4 months	NA	CORTICOBASAL SYNDROME	NA	Corticobasal degeneration (post-mortem exam): mild frontal and parietal atrophy, moderate-to-severe neuronal cell loss, and gliosis in the neocortex, basal ganglia, thalamus, and midbrain
Walerych et al., [99]	Female	79	Unilateral (Rt. hand)	posterior	Alien limb syndrome	NA	NA	NA	CORTICOBASAL SYNDROME	NA	NA
Menezes et al., [100]	Female	80	Unilateral (Lt. hand)	posterior	She verbalized a sensation that “my right hand does not belong to me”. She developed impaired fine-motor dexterity and ultimately lost the ability to use her right hand for routine functional tasks	Unremarkable	3 months	NA	CORTICOBASAL SYNDROME	NA	NA
Beltrao et al., [101]	Female	57	Unilateral (Lt. hand)	posterior	Her upper left limb seemed “forgotten”, with myoclonic jerks of the left hand	Brain MRI revealed disproportionate volumetric loss for age, more pronounced in the biparietal regions, worse on the left	2 years	NA	CORTICOBASAL SYNDROME	Donepezil 10 mg daily	Biparietal regions, mainly the left
McBride et al. [102]	Female	72	Unilateral (Rt. handed)	Frontal type	The right hand involuntarily grasped objects. No levitation, intermanual conflict, or mirror movements	MRI: Cortical atrophy in left parietal > frontal regions, bilateral caudate head volume reduction	NA	Patient is unable to stop involuntary movements, even with effort	Corticobasal syndrome (CBS)	Managed with strategies for grasping behavior	Parietal, frontal regions, caudate nucleus
Tilley et al., [103]	Male	64	Unilateral (Lt. hand)	posterior	An “alien” left arm with numbness and cramps	MRI showed significant cortical and hippocampal atrophy, cortical ribboning, and thalamic high signal on diffusion-weighted imaging	2 years	NA	corticobasal degeneration (CBD)	Donepezil 10 mg daily	Hippocampus, cortex, thalamus
Schaefer et al., [104]	Male	75	Unilateral (Lt. hand)	Frontal type	Rapid loss of control in left hand, stiffness, loss of fine motor skills; left hand grabbed his face involuntarily, intermanual conflict, transitive dyspraxia	MRI: increased and asymmetrical ventricles	NA	Used rthe ight hand to release the left-hand grip; controlled the hand at night by covering the arm and keeping the lamp on	Corticobasal degeneration, Parkinson’s syndrome	Restraint therapy, increased dopaminergic medication	Basal ganglia
Reyes et al., [41]	Female	76	Unilateral (Lt. hand)	Frontal type	Involuntary upper left limb movements for 2 h, having "a will of its own." Movements occurred intermittently for 7 days, with each episode lasting 10–20 min	MRI showed diffuse brain atrophy on T1 and extensive high signals in the periventricular white matter (leukoaraiosis) on T2/FLAIR. No hyperintensities are suggestive of stroke on DWI, and an intact corpus callosum on sagittal T1. MR angiography and EEG were normal	1 week	Levitation of the left hand with right-hand restraint	Diabetic hyperosmolar non-ketotic state with leukoaraiosis	Oral hypoglycemics	Diffuse brain atrophy
Chung et al., [56]	Female	66	Unilateral (Lt. hand)	Callosal type	Cross-localization of fingertips, cross-replication of hand postures, left ideomotor apraxia, left tactile anomia, left agraphia	MRI: Extensive callosal lesion (except rostrum)	NA	NA	DM, HTN	NA	Corpus callosum (body and splenium)
Sugawara et al., [105]	Female	72	Unilateral (Rt. hand)	Frontal type	Involuntary instinctive grasping reactions and compulsive manipulation of tools	MRI: high-intensity signal in left ACA region (genu to mid-body of CC). MRA: vessel occlusion of left A2. CT: no hemorrhage or exudate	5 months	Could restrain symptoms voluntarily by telling her hand not to move	ACA infarction	Self-restraint therapy	Frontal lobe, corpus callosum
Pooyania et al. [106]	Male	58	Unilateral (Rt. hand)	Frontal type	Lack of recognition of hand, involuntary movements, personification, self-restriction, impulsive grasping, groping, impaired coordination	Brain CT showed decreased attenuation in the left frontal lobe (non-hemorrhagic infarction in ACA territory). MRI confirmed a stroke in the left superior frontal gyrus. Brain angiogram: minor stenosis	6 weeks	The affected arm was forcibly braced against the counter. Once movements were extinguished, the task resumed	ACA infarction	Rehabilitation therapy (visualization, feedback, compensatory strategies)	Frontal lobe
Matsuyama et al. [107]	Female	62	Unilateral (Lt. hand)	Callosal-frontal type	Left hemiparesis, an involuntary writhing of the left hand, ideomotor apraxia, agraphia, neglect, executive dysfunction, and interhemispheric transfer dysfunction	Diffusion MRI: Hyperintensity in right frontal lobe convexity and corpus callosum. MRA: Right ACA occlusion	Persistent	Restrained involuntary movements of the left hand with the right hand	ACA infarction	Verbal-cue rehabilitation exercises	Right frontal lobe, corpus callosum
Alvarez et al., [59]	Male	66	Unilateral (Rt. hand)	Frontal, Callosal, Posterior types	Held right hand with left hand and concealed it under a blanket; right arm elevated and reached for objects involuntarily; difficulty feeding due to tremors; intermanual conflict, apraxia	MRI with diffusion-weighted imaging: Large ischemic infarction (left anterior cerebral artery); multiple lacunar infarctions	NA	Self-restraint; concealed right hand under the blanket	ACA infarction	Rehabilitation therapy, clonazepam trial	Left cerebral infarction
Hertza et al. [108]	Female	73	Unilateral (Rt. hand)	Post. type (sensory)	Unaware of using the wrong hand during tasks, such as reaching with the unintended hand. Finger-to-nose test showed the right hand unintentionally reaching the examiner’s finger	CT: Global cerebral atrophy; MRI: Acute infarct in left posterior MCA territory (~ 25% of region), clot in left M1 and M2 branches of MCA	Acute onset	Sitting on her hand to inhibit use; attempted visual-constructional tasks with effort and errors	MCA infarction	Visual-constructional tasks	Left parietal lobe
Yusoff et al., [109]	Male	62	Unilateral (Rt. hand)	Callosal type	Dysarthria, inability to swallow, follow orders, or raise left arm; 2 months post-stroke: AHS symptoms	CT: acute hypodensity in the right frontal lobe, right corona radiata, head of the right caudate nucleus, and right pons	4 months	The right hand counteracts all left-hand activities	MCA infarction, myocardial infarction	Oral amantadine 100 mg OD	Right frontal lobe, corona radiata, caudate nucleus, pons
Takenouchi et al., [110]	Female	9	Unilateral (Lt. hand)	Frontal type	Grabbing her hair involuntarily, paroxysmal, brief, stereotypical movements involving the left arm	MRI: T1/T2 hyperintensity and significant volume loss of the right hypothalamus	Unknown (lost follow-up)	Could terminate movements when instructed, but was unable to suppress initiation	Parry-Romberg syndrome	Observation of symptoms only, no pharmacological intervention	Right hypothalamus
Chokar et al., [40]	Female	36	Unilateral (Lt. hand)	Posterior type	Ideomotor apraxia of left hand; uncontrolled, non-purposeful movements described as “doesn’t go where you want it”, gripping involuntarily	MRI: Left cerebral hemisphere atrophy (alcohol abuse), subtle cortical thickening and signal alteration (right cerebral hemisphere and ipsilateral thalamus); CT angiogram: Hypodense region (right parieto-occipital); MRI (day 96): Atrophy (right temporal, parietal, occipital lobes)	6 months	Involuntary movements became less frequent with treatment	Parry-Romberg syndrome	IV methylprednisolone, cyclophosphamide, oral prednisolone, azathioprine	Right temporal, parietal, and occipital lobes
Moro et al., [111]	Female	47	Unilateral (Lt. hand)	frontal	Uncontrolled hand movements during routine tasks (e.g., grabbing objects, nose picking)	CT: Hemorrhagic damage in the frontal cortex, right cingulate gyri, paracentral lobule; white matter damage in the corpus callosum and anterior corona radiata	3 months	Used the right hand to restrain the left hand	Aneurysms in the cerebral arteries	Surgery for an aneurysm	Hemorrhagic damage in the frontal cortex and associated structures
Panda et al., [112]	Male	68	Unilateral (Lt. hand)	frontal, posterior	Sudden onset of abnormal involuntary movements of the left upper limb. While relaxing after dinner, he felt that his right hand was being touched by someone else. Upon looking, he saw his left upper limb groping and trying to hold his right hand. He could not perceive his left upper limb as his own. Abnormal levitation of the left upper limb occurred. Despite recognizing the left side of his body, he experienced disowning of the left upper limb	MRI of the brain revealed altered signal intensity in the right frontoparietal region (hypointense on T1-weighted and hyperintense on T2-weighted/FLAIR sequences) with swelling of adjacent gyri, suggesting an acute infarct	2 h	Feeling of unfamiliarity persisted, though he could control some movements	Right frontoparietal infarction	Antiplatelet medication and statin	Right frontoparietal lobe
Pouget et al., [113]	Male	37	Unilateral (Lt. hand)	callosal	The left arm displayed counteracting movements against the right hand’s actions (e.g., “undoing what the right hand accomplished”). The patient and family reported “thief hand” behavior, such as taking back a plate or money after the right hand placed them. The patient overcame these movements by interrupting right-hand movements, wrestling with the left hand, or sitting on it	MRI revealed a corpus callosum lesion (rostrum, genu, and body affected; sparing the splenium)	2 years	The patient used strategies like wrestling with the left hand, making a special concentration effort, or sitting on the left hand	Rupture of an anterior artery aneurysm	NA	Corpus callosum
Sabrie et al., [114]	Male	25	Unilateral (left hand)	Posterior type	Left hand controlled by “someone else”; painful dystonia of left arm; cramp-like feeling, abnormal bending of elbow, and fisting of hand	Diffusion-weighted MRI: ischemic lesion in the right posterior cerebral territory	1 week	Aggravated by walking/writing; relieved by rest and sleep	Mood disorder, left hemiparesis	Clonazepam initially ineffective; resolved with carbamazepine 200 mg BID	VPL of the right thalamus, medial temporal lobe
Liu et al. [115]	Male	45	Unilateral (Rt. hand)	Posterior type	Startled by the right hand entering the visual field, unaware. Resolved spontaneously upon hospital arrival	MRI: Small embolic-like events in left MCA territory, parietal lobe (DWI and FLAIR sequences)	~ 1 h	Resolved spontaneously upon arrival at the hospital	Transient ischemic attack (TIA)	NA	Parietal lobe
Kim et al., [116]	Male	48	Unilateral (Rt. hand)	Callosal type	Difficulty controlling the right hand without volition, interrupting the left hand's action (e.g., drying face with a towel); diagnostic apraxia due to affection of the posterior end of the corpus callosum	MRI: Extensive multifocal encephalomalacic changes in left temporo-parietal occipital lobe and both frontal lobes; hemorrhagic residual changes from old traumatic lesions	NA	NA	Traumatic intracranial hemorrhage, right-sided hemiplegia, epilepsy	NA	Corpus callosal thinning (disconnect syndrome)
Lunardelli et al., [34]	Female	47	Unilateral (right) with left-hand apraxia	Callosal type	The right hand did not respond or write as intended; the left hand interfered with activities of the right hand; tactile anomia; the patient was aware of errors but unable to correct them	MRI: Confluent white matter lesions; severe corpus callosum involvement (caudal portion); T1-weighted: Hypointense lesions (black holes)	NA	Aware of unwanted movements but unable to inhibit them	MS (35 years), family history of IBD	NA	Caudal portion of the corpus callosum
Le et al., [44]	Male	88	Unilateral (Lt. hand)	Posterior type	Intermittent involuntary movements of the left forearm and hand (e.g., slapping himself while napping)	MRI: Acute infarctions (right temporal lobe, right parietal cortex, right parietal subcortex)	1 day	Hand movement spontaneously improved after one day	Diastolic dysfunction, atrial flutter (suspected thromboembolism)	Resolved spontaneously	Right parietal cortex
Shao et al., [60]	Male	57	Bilateral	Callosal type	Intermittent episodes of lack of control in either hand (e.g., unintentionally using both hands to grab an object or turning a steering wheel in opposite directions); unable to prevent or correct errors	MRI: Abnormal signals in the corpus callosum (sagittal view); T1: Hypointensity; T2, FLAIR, DWI: Hyperintensity; Enhanced MRI: Hyperextension in the corpus callosum; consistent with Marchiafava-Bignami disease	2 months	Treated with high-dose vitamin B1 for one month; improved	Marchiafava-Bignami disease	High-dose vitamin B1	Corpus callosum
Hosokawa et al. [61]	Male	80	Unilateral (Lt. hand)	Post. type	Perceived a “third left leg” with involuntary left-hand movements, occurring when in bed and drowsy	MRI: Hemorrhagic foci in the right dorsolateral brain (pontine tegmentum to lower midbrain tegmentum); lesions spread to medial lemniscus and locus coeruleus	NA	NA	Supernumerary phantom limb levitation (SPL), pontine hemorrhage	NA	Pons
Gheewala et al. [75]	Male	67	Unilateral (Rt. hand)	Post. type	Difficulty in bilateral hand coordination, right-hand paresthesia, and intermittent involuntary touching of his face with normal grip strength	CT: Chronic right PCA infarct in occipital and temporal lobes; MRI: Acute left inferior parietal lobe infarct including postcentral gyrus	One day	NA	Chronic atrial dysrhythmia, NSTEMI, and acute ischemic stroke	Resolved spontaneously	Left inferior parietal lobe
Gellman et al. [117]	Female	13	unilateral	Callosal type	Fever, headache, vomiting, odd behaviors such as involuntary throwing of objects, and involuntary movements	MRI-DWI: Lesion in the posterior aspect of the splenium of the corpus callosum, suggestive of acute ischemia	3 weeks	The patient reported involuntary actions	Small PFO	Resolved spontaneously	Corpus callosum
Terazzi et al., [118]	Male	89	Unilateral (Lt. hand)	posterior	His left limb displayed levitation episodes, grasping objects ataxically and clumsily, despite acknowledging the limb as his own. Complaints included involuntary movements during periods of low attention, e.g., twilight or fatigue, and self-harming behaviors misattributed to external causes	CT and MRI revealed ischemic lesions in the right thalamus and calcarine cortex	2 weeks	NA	NA	Three drugs for sleep (trazodone, mirtazapine, and clonazepam) did not alter symptoms	Right thalamus and calcarine cortex
Murdoch et al., [119]	Male	74	Unilateral (Rt. hand)	posterior	Involuntary chaotic movements of the right arm, including slapping, grabbing objects, and strangling himself. Recognized the arm as his own but out of control. Needed assistance to release strangulation holds	Large left subacute hemorrhagic infarct in the posterior parietal and occipital cortices, with likely involvement of the splenium of the corpus callosum	2 weeks	Therapy involved focused attention on the affected arm, visual scanning techniques, and distraction during personal care tasks	Large left subacute hemorrhagic infarct affecting the posterior cerebral artery territory	Therapy included visual and attentional strategies	Infarct in the left posterior parietal and occipital cortices; likely splenium of the corpus callosum
Verleger et al., [120]	Male	69	Unilateral (Lt. hand)	callosal	Left-hand counteracts the right-hand (e.g., closing doors when the right hand opened them)	MRI: Lesion in the corpus callosum (genu to posterior isthmus); leukoaraiosis due to hypertensive microangiopathy	4 months	NA	Infarction of the left arteriole cerebri pericallosa	NA	Corpus callosum

## Clinical manifestations of alien hand syndrome

Alien Hand Syndrome (AHS) refers to apparently purposive, involuntary movements of a limb that the patient claims not to have initiated consciously. It manifests as distinct clinical syndromes, which often correlate with specific neuroanatomical damage:

The callosal subtype typically involves the non-dominant hand and is characterized by intermanual conflict, where one hand interferes with the actions of the other. This phenomenon is thought to arise from a failure to inhibit the non-dominant hemisphere during tasks governed by the dominant hemisphere [9]. The frontal subtype, usually involving the dominant hand, is associated with lesions in the supplementary motor area (SMA), sometimes extending into the corpus callosum. It is marked by compulsive grasping, involuntary object use, and “utilization behavior” where patients inappropriately manipulate tools or objects in their environment, even when the action is contextually irrelevant [9]. A posterior variant has also been described, involving lesions in the parietal or occipital lobes. This form presents with limb levitation, non-goal-directed writhing finger movements, and a sense of estrangement from the limb. These symptoms may mimic other movement disorders but are distinct in their lack of volitional drive [5].

The dual premotor system hypothesis may explain these behaviors. Internally initiated movements are mediated by the medial premotor system (SMA and cingulate gyrus), whereas externally triggered responses involve the lateral premotor cortex (PMC). Lesions to the SMA can result in PMC disinhibition, leading to reflexive grasping and other stimulus-bound motor behaviors [13].

Transient AHS (TAHS) is often seen with isolated callosal lesions, while chronic AHS more commonly arises from combined fronto-callosal damage, highlighting the importance of lesion localization [14]. Recent studies have expanded the clinical spectrum of AHS, underlining the importance of recognizing posterior involvement and atypical presentations [5].

## Pathogenesis of alien hand syndrome

Cerebral insults, such as post-surgical trauma, neoplasms, vascular events, and neurodegenerative diseases, are associated with the pathophysiology of AHS. Vascular risk factors (e.g., hypertension) may contribute to strokes that precipitate AHS, but AHS itself is primarily a consequence of structural brain lesions. [15]. The thalamus, corpus callosum, anterior cingulate gyrus, posterior parietal cortex, supplementary motor area, and anterior prefrontal cortex are frequently affected, leading to both motor and sensory abnormalities. Agnostic dyspraxia is a motor disorder that may lead right-handed people to exhibit conflicting motions with their left hand. The perception of extra limbs or the feeling that the hand is strange are examples of sensory symptoms [16]. The frontal-parietal network is assumed to mediate the dynamic, multisensory picture of one’s body that enables smooth interaction with the environment, and AHS may be a sign of a disturbed body schema [17]. There are two subtypes of AHS: frontal and callosal. Damage to the medial prefrontal cortex causes the frontal subtype, which is characterized by involuntary grabbing and groping, whereas corpus callosum damage causes the callosal subtype, which mostly shows up as inter-manual conflict [10]. More frequently, the right cerebral hemisphere is implicated, with motor and sensory unawareness resulting from damage to the right parietal cortex [18]. Due to a disturbance in inhibitory control, injuries to the frontal lobe, specifically in the cingulate gyrus and supplementary motor region, have been connected to uncontrollable motor activities [6]. Callosal AHS is comparatively uncommon because of the corpus callosum’s strong vasculature, but when it does happen, it frequently coexists with other brain injuries, making diagnosis difficult [19]. Comprehensive neuroimaging is also essential for diagnosis because AHS can present with a wide range of neurological symptoms [20].

## Imaging/neuroanatomy

**Fig. 4:**
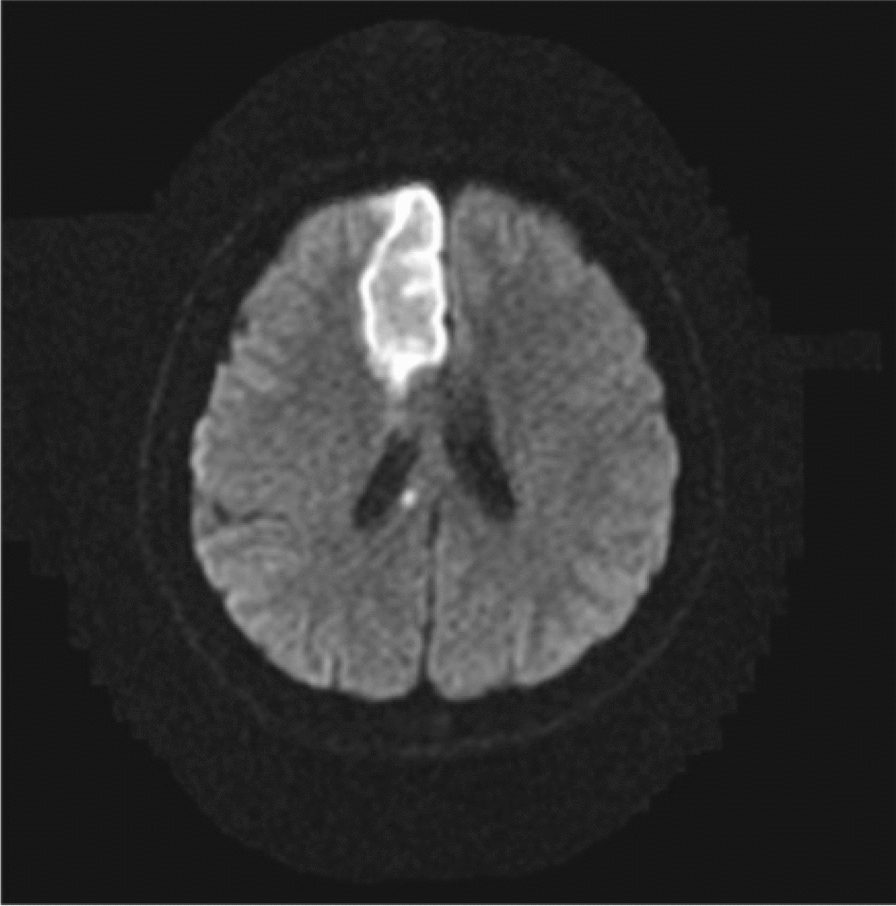
Diffusion-weighted magnetic resonance image showing acute infarction in the right frontal lobe & adjacent corpus callosum. [43] Copyright © 2012 by Korean Academy of Rehabilitation Medicine

**Fig. 5:**
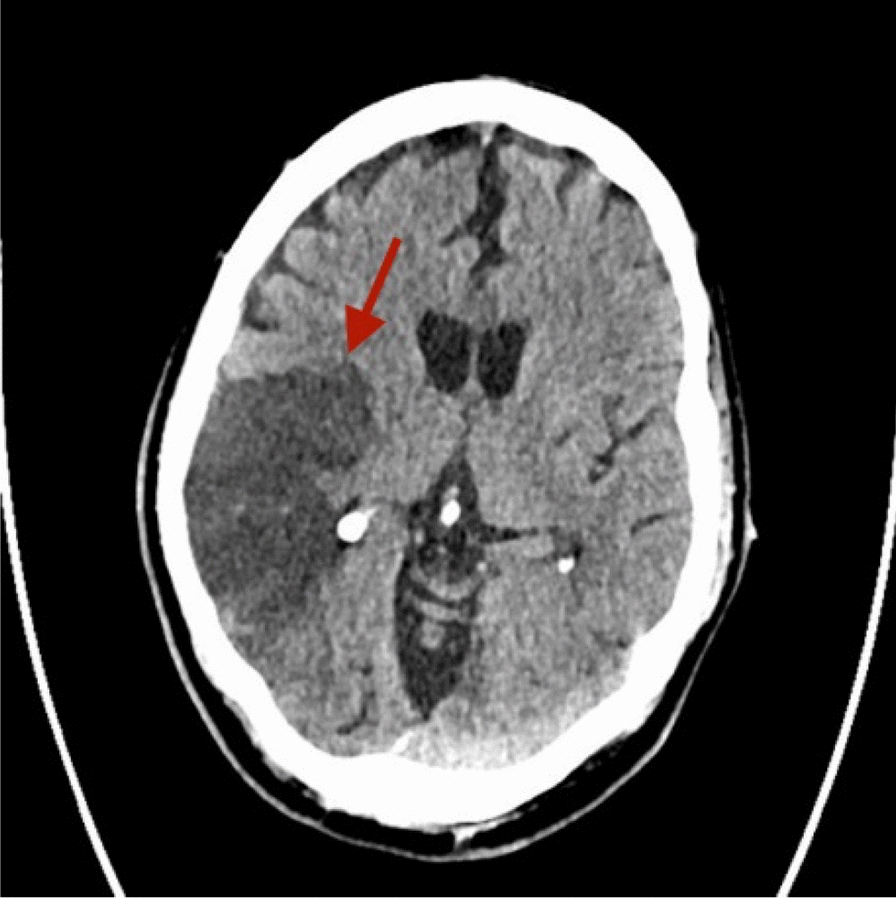
Non-contrast CT scan of right middle cerebral artery (MCA) territory (temporoparietal region). [42] Copyright © 2024, Rashwan et al.

**Fig. 6:**
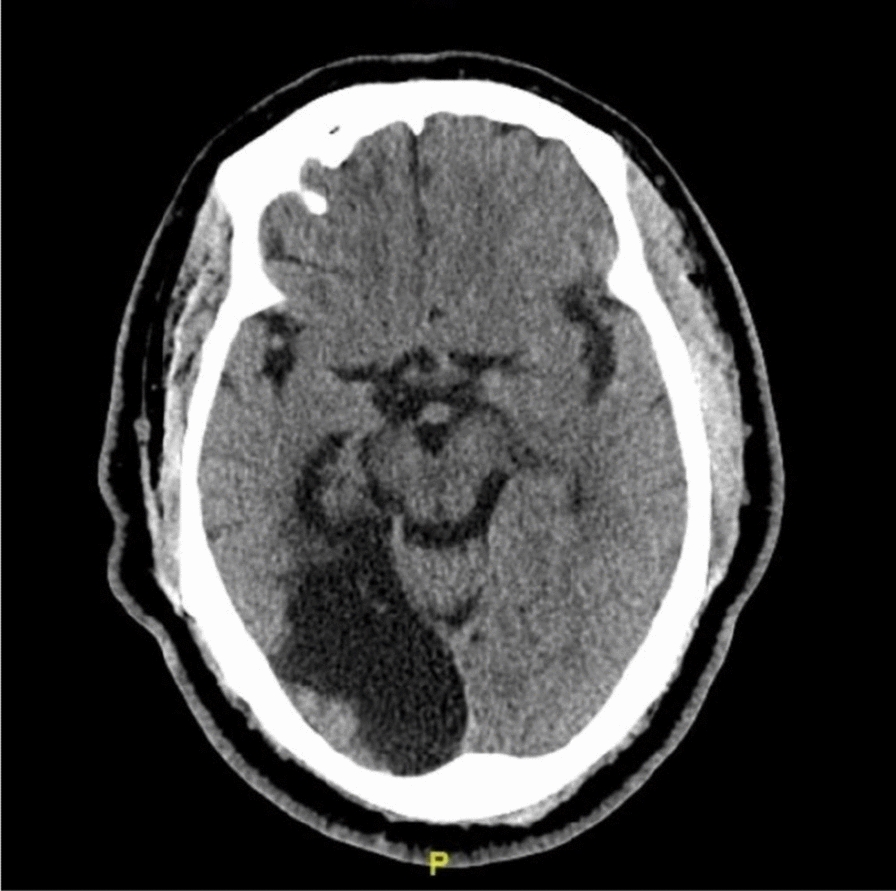
CT scan of old posterior cerebral artery (PCA) territorial infarct involving occipital & temporal lobes.[75] Copyright © 2019, Gheewala et al.

Alien limb syndrome is a neurological disorder with neuroanatomical pathophysiology best described in subtypes (Table 2 and 3).The frontal variant (Fig. 4) affects the function of the dominant upper hand, and the patient experiences disruption in manual movements, especially in grasping and releasing tasks. Voluntary movements of the limbs are absent in the patient. This variant is related to lesions in the frontal lobe of the dominant hemisphere, prefrontal cortex, and supplementary motor area [5, 42]. The callosal variant impacts the non-dominant hand with movement disorder, apraxia, and neglect because of damage to the corpus callosum [43]. The posterior variant also occurs in the non-dominant hand, but the patient feels that the affected limb is foreign to his body; that is, he is unable to voluntarily control the movements of the affected limb, linked with non-dominant parietal and temporal lobe damage (Figs. 5 and 6)[5, 44].

**Table 2:** Anatomical Variants of Alien Hand Syndrome

Variant	Lesion in the Brain	Signs and Symptoms	Common Cause(s)	References
Frontal (Fig. 1)	Brodmann area 6 (supplementary motor area), Brodmann areas 24, 25, 32, and 33 (cingulate gyrus), corpus callosum	Groping, grasping, and releasing of hands, utilization behavior, disruption of manual movements, loss of voluntary movements	Tumor, infarction of the anterior cerebral artery, trauma	[10, 18, 21, 22]
Callosal (Fig. 2)	Corpus callosum	Intermanual conflict and strong grip syndrome, movement disorder, apraxia, neglect of the non-dominant hand	Stenosis of the basilar artery, callosotomy, and tumor	[10, 15, 18, 21, 22]
Posterior (Fig. 3)	Parieto-occipital cortices, thalamus	Levitation, writhing fingers, and involuntary movement of the non-dominant hand	Infarction of the posterior cerebral artery	[22, 23]

**Table 3:** Pathological variants of alien hand syndrome

Etiology	Examples	References
Neurodegeneration	Corticobasal syndrome (CBS), Corticobasal clinical overlap syndromes (with PSP, posterior cortical atrophy, primary progressive aphasia, multiple system atrophy, dementia with Lewy bodies), CBS with PSP pathology, Alzheimer’s disease, progressive dementia (not otherwise specified), thalamic dementia	[6, 24, 25]
Stroke	Frontal lobe infarct (contralateral medial), anterior communicating artery stroke/rupture, corpus callosal infarct/hematoma, intracranial hemorrhage, subdural hematoma, parietal infarct, thalamic infarct, occipital infarct	[24, 26–30]
Prion Disease	Sporadic Creutzfeldt-Jakob disease, familial Creutzfeldt-Jakob disease	[31, 32]
Tumors	Midline tumors, astrocytoma, oligodendroglioma	[6, 33]
Demyelination	Multiple sclerosis, Marchiafava-Bignami disease, progressive multifocal leukoencephalopathy, hereditary diffuse leukoencephalopathy with spheroids	[6] [34] [35]
Iatrogenic	Corpus callosotomy, electrical cortical stimulation, radiation treatment of oligodendroglioma, resection of frontal lobe tumor	[36–38]
Seizures	Epilepsia partialis continua, ictal event	[36] [39]
Developmental	Parry-Romberg syndrome	[40]
Miscellaneous	Migraine aura, posterior reversible encephalopathy syndrome, spontaneous pneumocephalus, diabetic hyperosmolar non-ketotic state with leukoaraiosis	[31, 32, 41]

AHS arises from several cerebral insults, such as trauma, surgery, tumors, strokes, and progressive neurodegenerative diseases. The frequently affected regions are usually the prefrontal cortex, parietal cortex, supplementary motor area, cingulate gyrus, and corpus callosum [10].

**Fig. 7:**
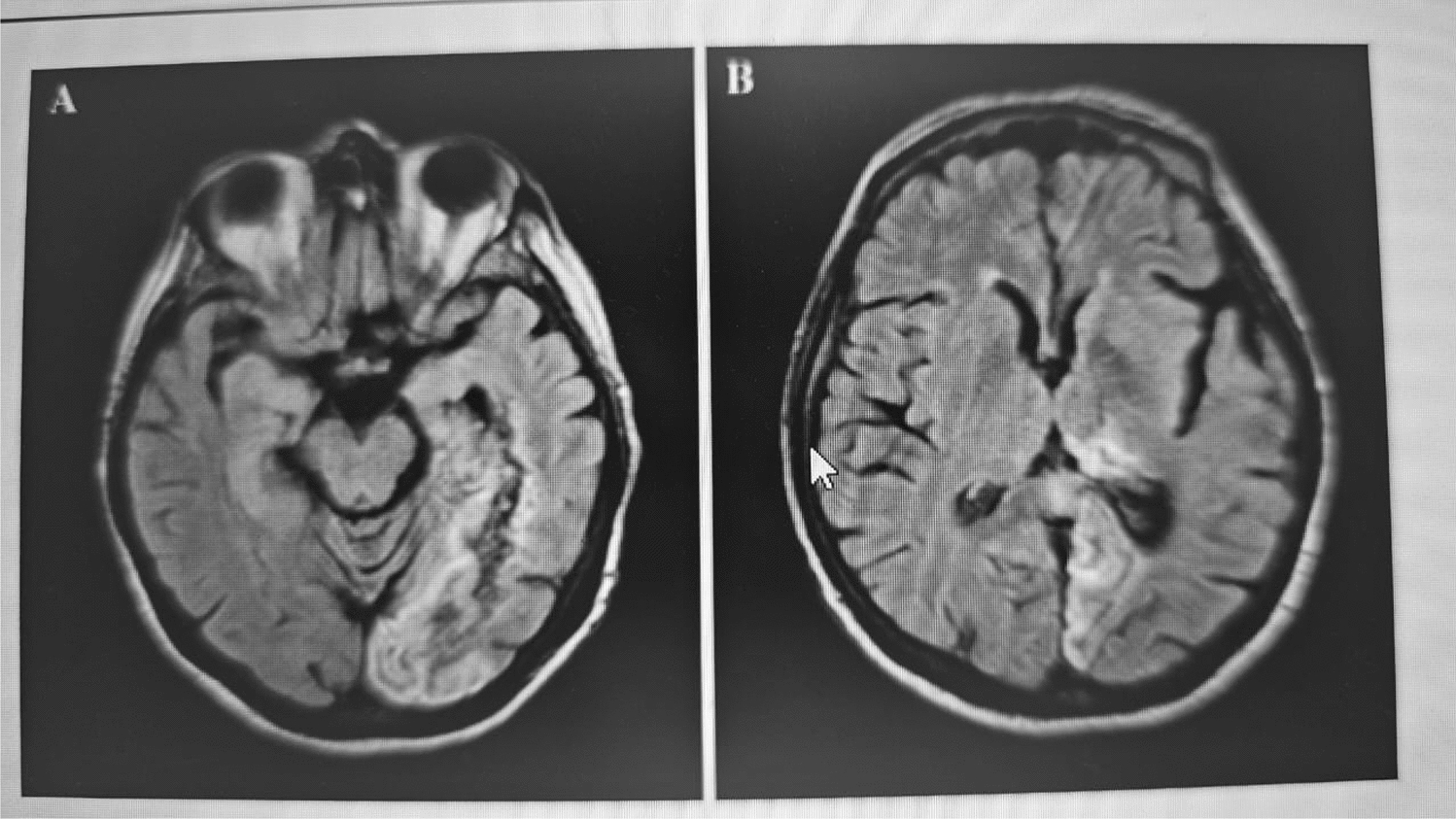
Follow-up FLAIR MRI of left posterior cerebral artery (PCA) territory: gliotic changes in temporo-occipital region (**A**) & posterior thalamus (**B**); FLAIR: Fluid Attenuated Inversion Recovery [46]

Frontal form is usually associated with left hemisphere dysfunction, which affects the right hand. In contrast, callosal and posterior forms are commonly associated with right hemisphere strokes resulting from anterior cerebral artery and posterior cerebral artery territory infarction, which affects the left hand. There can also be mixed forms of AHS involving features of both frontal and callosal forms. Moreover, the legs can also be affected [45]. The posterior variant can be caused by an infarct in the left subcortical posterior cerebral artery territory. MRI (Fig. 7) showed ischemic lesions in the posterior part of the thalamus, parahippocampal gyrus, temporal lobe posterior parts, and the corpus callosum’s lateral part. The thalamic involvement was responsible for the involuntary movements and the sense of estrangement of the affected hand [46].

**Fig. 8:**
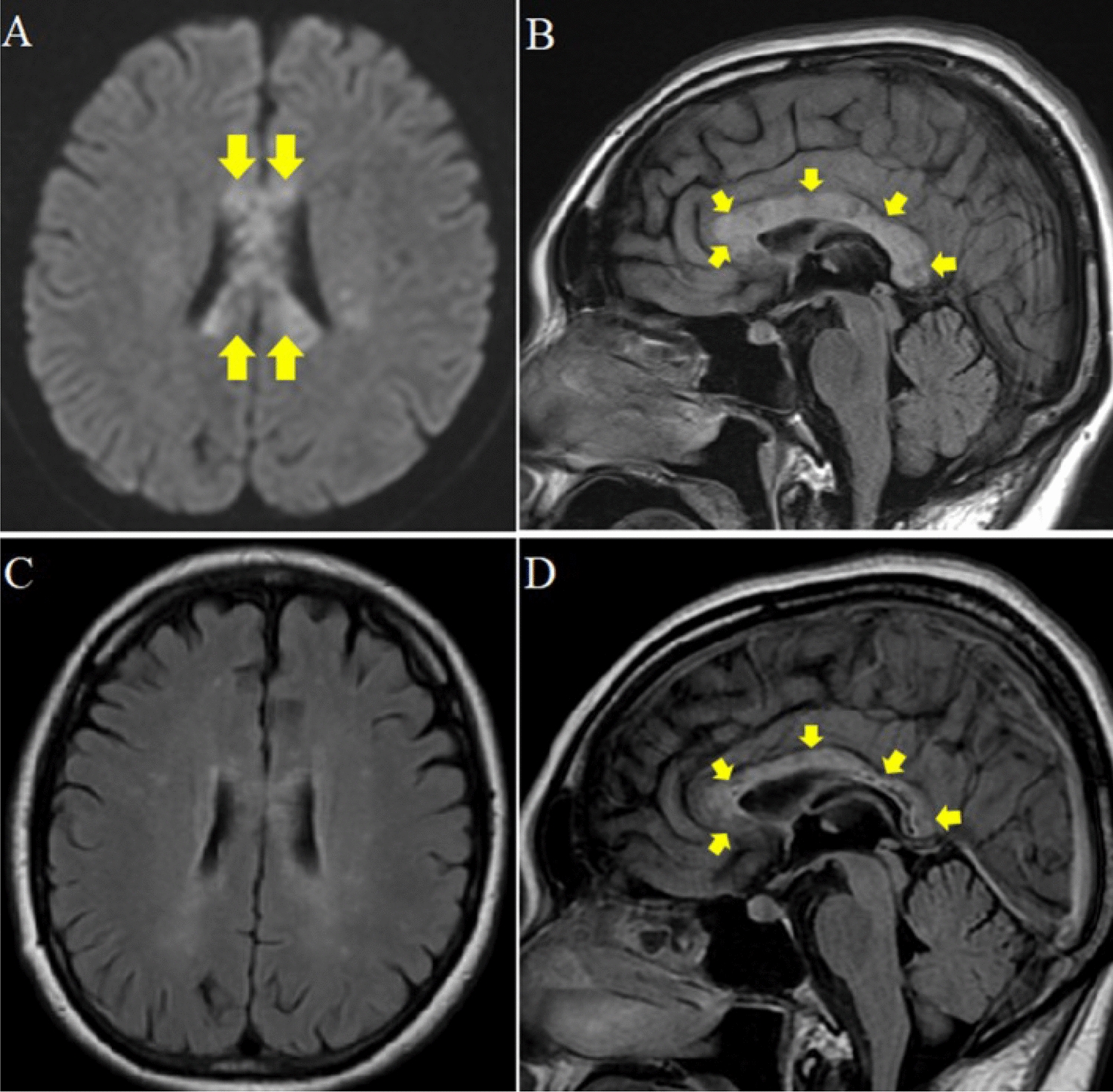
Splenial corpus-callosum lesions on DWI (patchy high signal (**A**)) & FLAIR (edema & regression (**B**)), arrow indicates irregular signals and low intensity at the core; FLAIR: Fluid Attenuated Inversion Recovery, DWI: Diffusion-weighted imaging. [20] Copyright © 2018 Shozawa et al.

A case study by Shozawa et al. (2018) depicted a patient who presented with difficulties in walking and involuntary movements in the left hand that the patient did not control. A thorough neurological examination revealed disruption of the corpus callosum [20]. The brain MRI showed high intensity in the corpus callosum with an edematous and irregular-intensity core (Fig. 8). This callosal variant impacts the non-dominant hand with movement disorder, apraxia, and neglect because of damage to the corpus callosum.

**Fig. 9:**
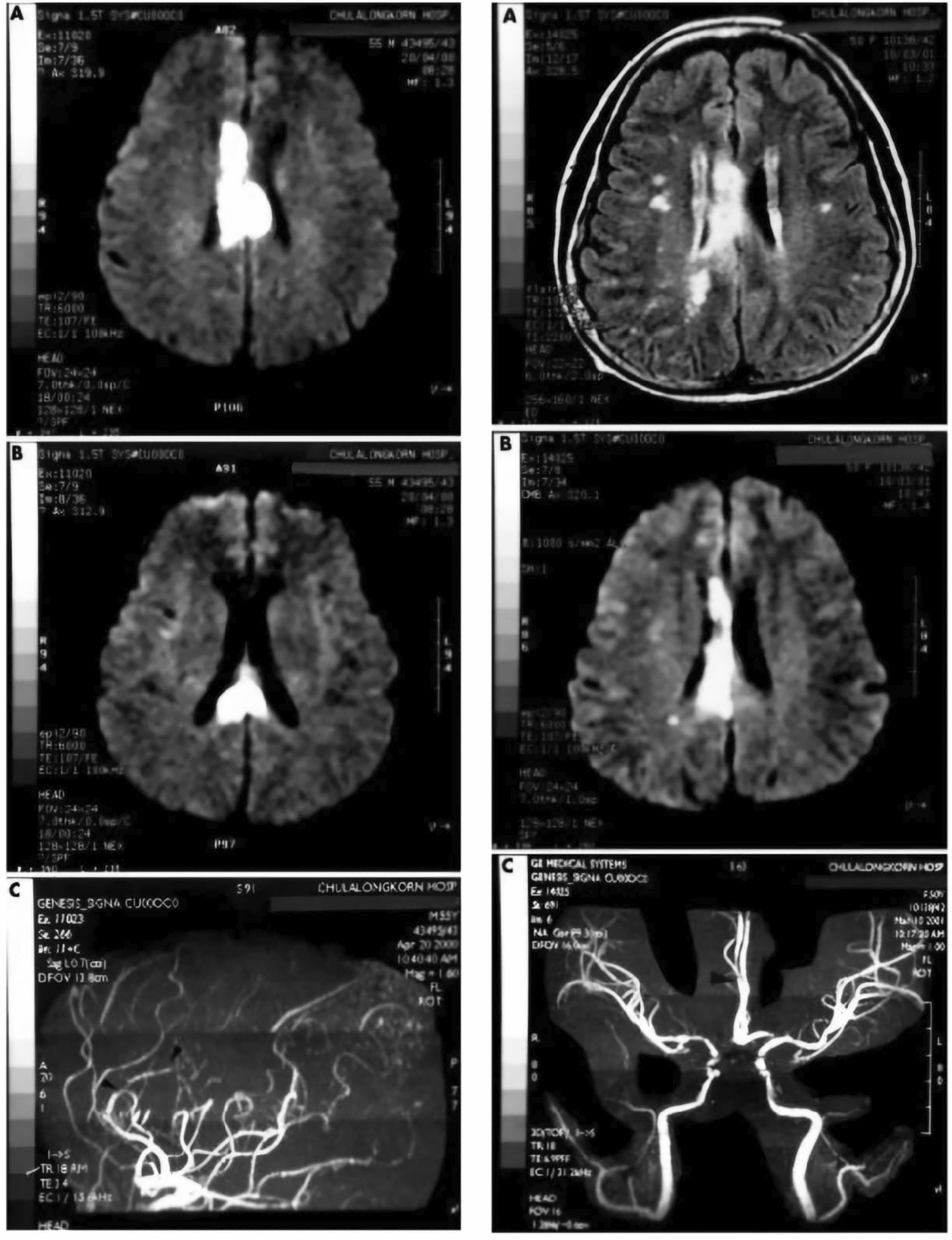
FLAIR/DWI showing Corpus callosum infarction (**A**-**B**) and magnetic resonance angiography showing pericallosal artery narrowing indicated by arrow (**C**); FLAIR: Fluid Attenuated Inversion Recovery, DWI: Diffusion-weighted imaging. [47] Copyright © 2001 N C Suwanwela, N Leelacheavasit

N. Suwanwela & Leelacheavasit et al., 2002, published a case where the patient was diagnosed with AHS and presented with symptoms of the sensation of an extra hand touching his left hand and his left hand acting independently of its own accord [47]. The brain MRI findings (Fig. 9-Left) showed lesions in the body and splenium of the corpus callosum, which were consistent with an infarct. MRA revealed a unique branching of the anterior cerebral artery with a single trunk for the pericallosal branches (Fig. 9-Right), which supplies the corpus callosum, and it showed evidence of narrowing and stenosis [5].

A comprehensive literature review was conducted by Lawson McLean et al. 2022 [4] in, to examine clinical presentations, treatment approaches, and radiological findings associated with alien hand syndrome (AHS) occurring after aneurysmal subarachnoid hemorrhage (SAH). This analysis revealed 17 reported cases. Among these, aneurysms of the anterior communicating artery were the most frequent source of SAH, accounting for 10 out of 17 cases. Pericallosal artery aneurysms followed, contributing to 7 out of 10 cases.

**Fig. 10:**
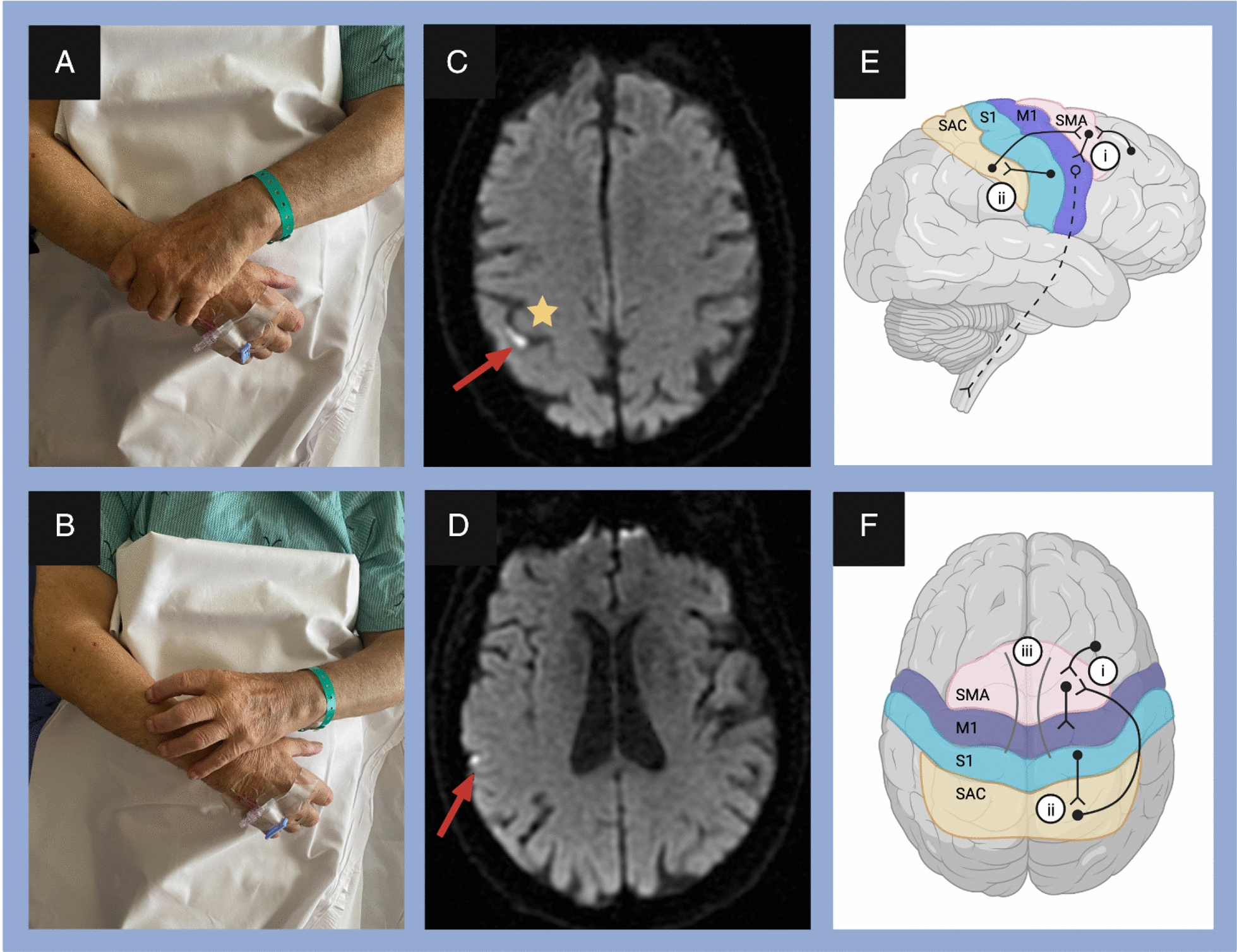
The patient imitated motor behaviors during the event, including grabbing the right wrist (**A**) and scratching the right arm (**B**) with the left hand when the patient attempted to put his left arm down. Diffusion-weighted imaging (DWI) axial view shows infarcts (red arrows) in the right parietal near the hand knob area in the motor (precentral) gyrus (yellow star) (**C**) and right temporal lobe (**D**). Diagram showing possible sensorimotor regions and pathways involved in alien hand syndrome in sagittal (**E**) and axial (**F**) views. [48] Copyright © 2023, Cambridge University Press

A case report of alien hand syndrome by Dmitriew et al., 2024, showed several small acute cortical infarcts (Fig. 10) [48]. This corresponds to the posterior variant, which occurs in the non-dominant hand, and the patient feels that the affected limb is acting of its own accord with a mind of its own, as seen in this patient who reported abnormal drifting up of the hand beyond his control.

It was found in a study that damage to the supplementary motor area, which is involved in initiating and planning movements, can lead to loss of inhibition of movements, thus leading to the involuntary movement of the hand [49]. Damage to the communication and coordination center, the corpus callosum, which links the two parts of the brain, could lead to desynchronization and dissociation of the movements of the hand. This causes the hand to act independently on its own, thus leading to a sense of detachment of the hand from the rest of the body, with the hand acting as if it has a mind of its own [5]. The parietal lobe is involved in motor planning and the integration of sensory information. Thus, any damage can lead to the impairment of the patient’s ability to perceive his hand movements, leading to a disconnect between intention and action [50].

Bahji et al. (2022) highlighted the appearance of AHS in a patient with diagnosed Lewy Body dementia. The patient presented with conflicting independent actions of the affected limb. LBD was confirmed by loss of dopamine transporter in MRI and PET. Imaging revealed the involvement of the primary motor cortex, premotor cortex, and angular gyrus in the aberrant movements observed in AHS [51].

**Fig. 11:**
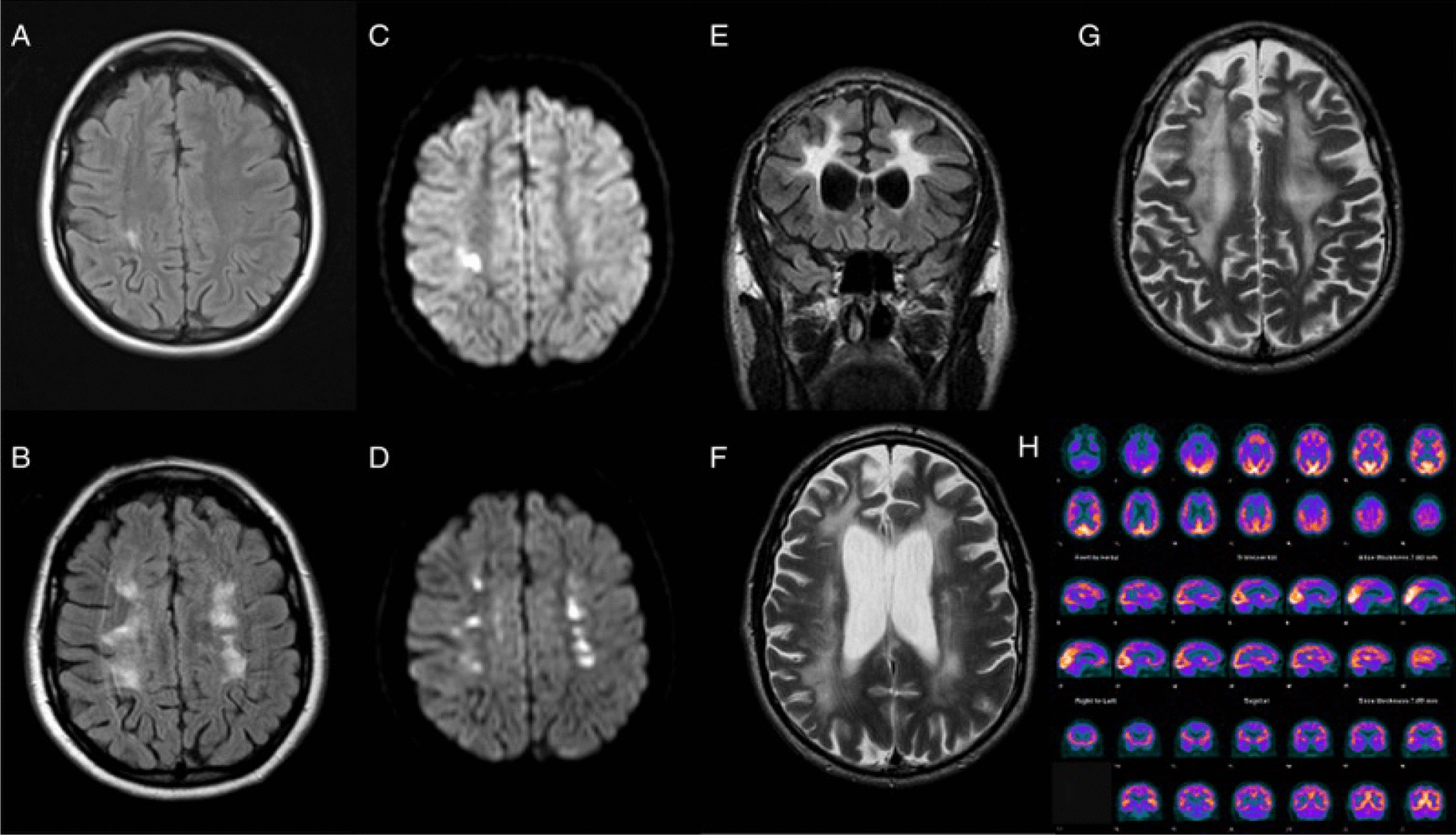
Serial FLAIR/DWI (**A**–**E**), T2- T2-weighted imaging (**F**-**G**), and FDG-positron emission tomography (**H**) demonstrating changes as seen in neurodegenerative AHS; FDG: Fluorodeoxyglucose [53]

AHS was also found to be related to hereditary diffuse leukoencephalopathy with spheroids, which is a rare genetic disorder. The patient in a case reported by Dongre et al. (2022) presented with symptoms of progressive rigidity, dementia, involuntary left arm levitation, and mirror movements, which are characteristic of posterior alien hand syndrome [52]. Brain imaging (Fig. 11) revealed extensive parietal white matter abnormalities, including significant volume loss and signal changes in the corpus callosum [53].

Carrazana et al. (2001) reported a case involving a woman with seizures who had an unusual presentation of AHS during seizures. She had a brain tumor with frontal lobe involvement. Neuropsychological testing showed psychopathological markers corresponding to the tumor location in the mesial frontal area. It also became apparent that the symptoms of the alien hand syndrome were not seen between seizures, so it was likely secondary to seizure activity. As earlier postulated, it was believed that epileptic seizures interfered with neural pathways between both sides of the brain, causing disorganization of the normal motor functions, hence producing the movements that characterize the condition [54].

**Fig. 12:**
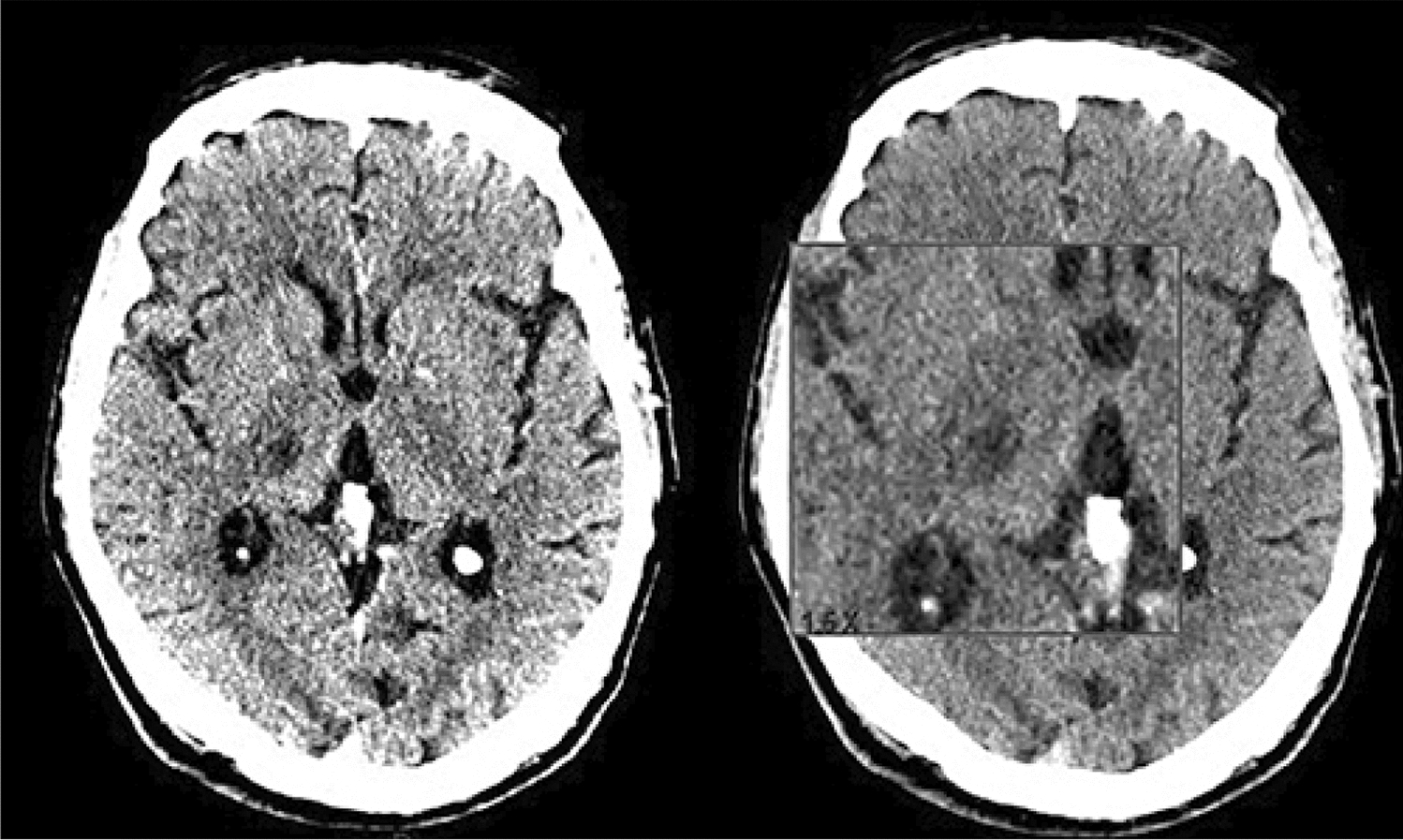
24-h computed tomography brain scan showing a new-onset right thalamic ischemic lesion. [55] Copyright © 2020, Silverchair Publisher

Russo et al. (2020) reported a case of a patient who presented with trauma to the eye due to movement disorders and was found to have segmental ataxia, weakness in his limbs, and mild paresis, all indicating ischemic lesions in the brain. A CT scan showed an ischemic lesion in the right thalamus (Fig. 12). Such thalamic infarcts can be caused by lesions in the small lenticulostriate vessels supplying it, and they usually produce a posterior variant of AHS. Thalamic infarct can cause frontoparietal disconnection and limb sensory deficit due to lesions in the somatosensory nuclei (ventral posteromedial and posterolateral) located within the thalamus [55].

Lesions in the supplemental motor area, cingulate gyrus, and corpus callosum result in the anterior variant, which is mainly caused by tumors, infarction, or trauma. Lesions resulting from callosotomy, tumor, or infarction in the corpus callosum cause callosal variants.Creutzfeldt–Jakob disease, corticobasal syndrome, or infarction of parieto-occipital cortices and thalamus cause the posterior variant [22].

**Fig. 13:**
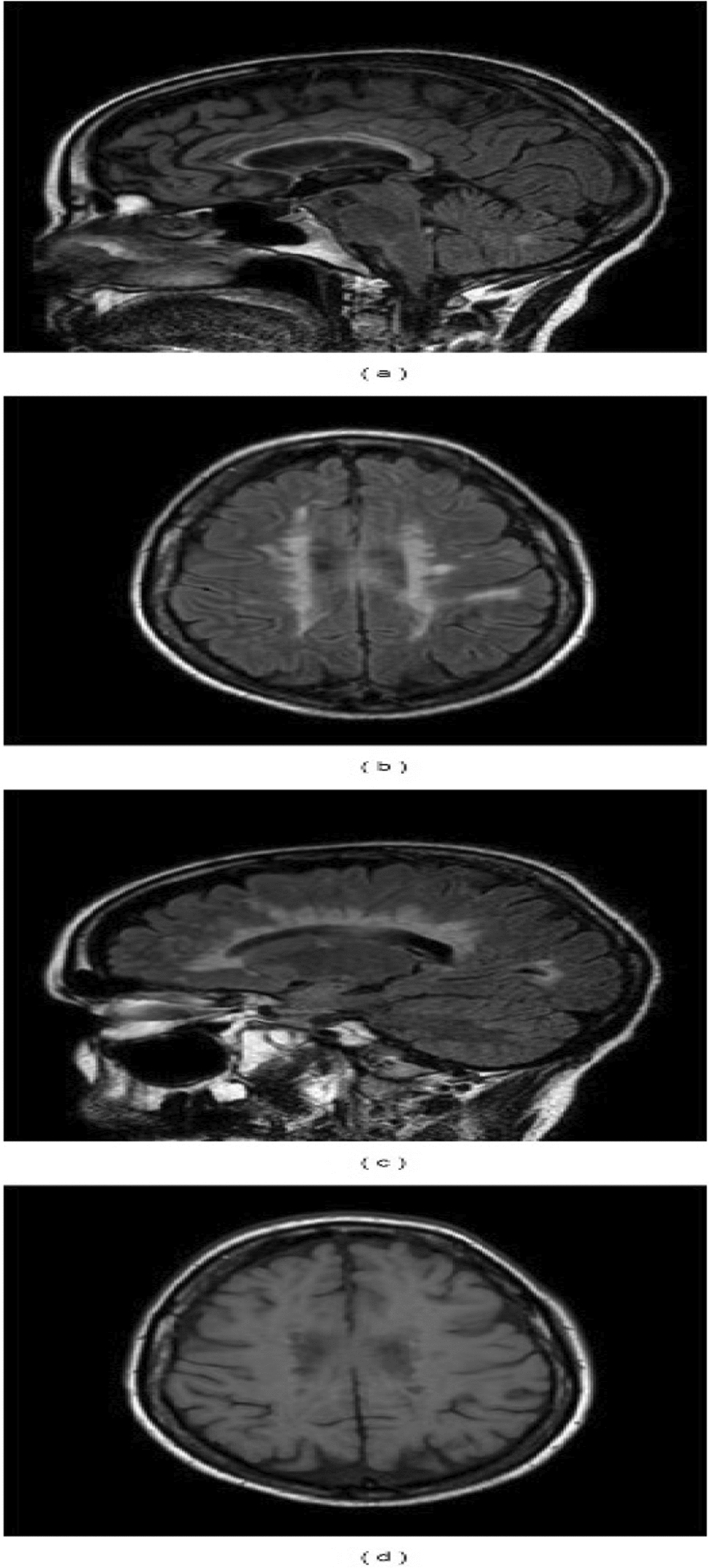
Sagittal (**a**), axial (**b**), and parasagittal (**c**) FLAIR MRI scans showing corpus callosum involvement. T1 axial scan (**d**) shows the presence of numerous T1 hypointense lesions (“black holes”). [34] Copyright © 2014 A. Lunardelli et al.

**Fig. 14:**
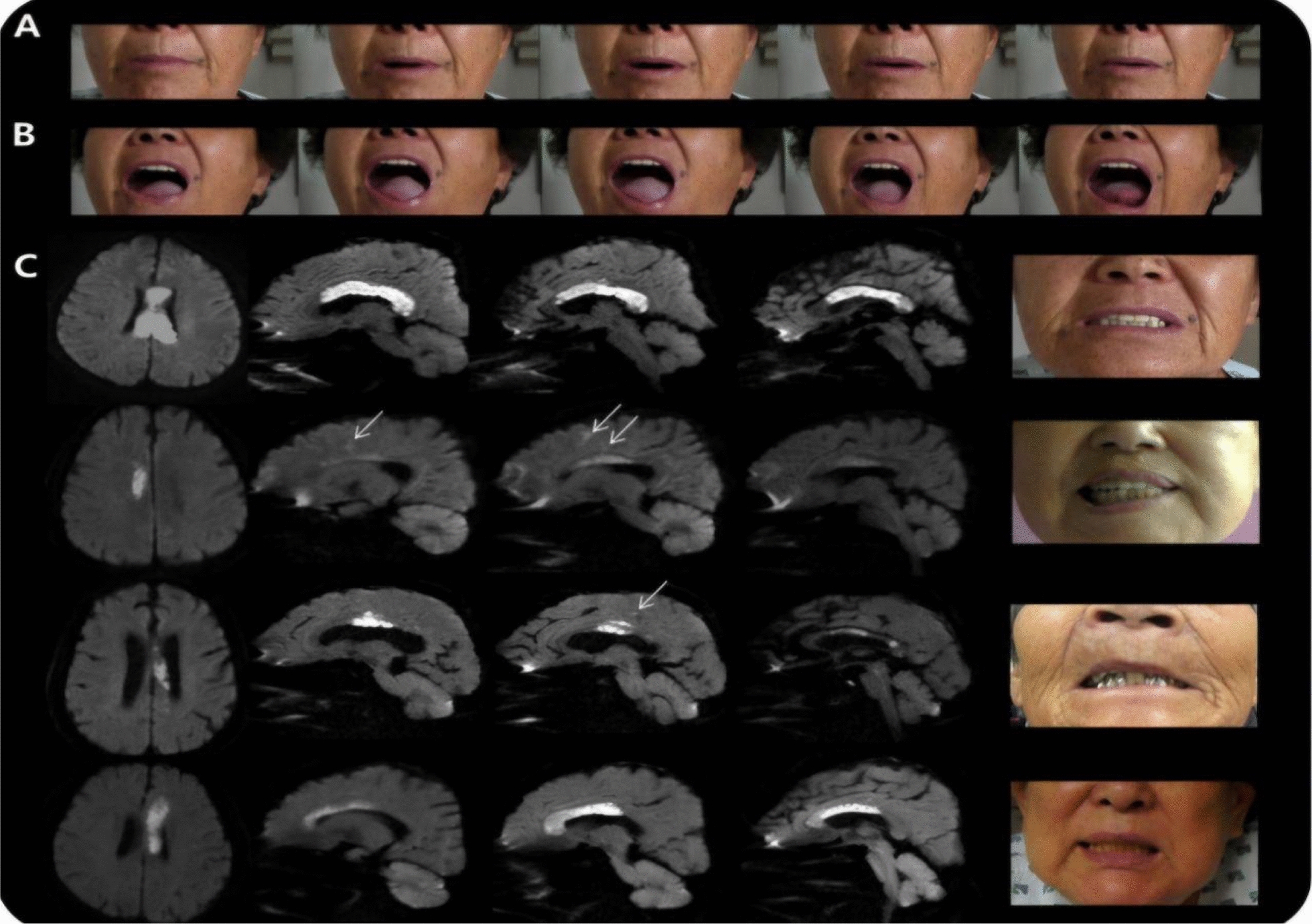
Facial-labial movement asymmetry in callosal AHS: sequential clinical photos (**A**–**C**) with corresponding diffusion MRI lesions (arrows) [56]

AHS is also infrequently seen in multiple sclerosis due to its involvement in the corpus callosum. Its clinical manifestations are different from the common callosal variant and present with bilateral involuntary hand movements, callosal apraxia, and unilateral agraphia. Certain alien hand behaviors have been linked to increased fatigue and anxiety, and these are all symptoms prevalent in MS, thus indicating a connection between MS and AHS. Unilateral agraphia is a classic symptom of interhemispheric disconnection (Fig. 13), which could be attributed to the disruption of communication between the left hemisphere’s centers and the right hemisphere’s motor areas, which control hand movement [34]. Callosal lesions were found to correlate with asymmetrical orolingual movements (Fig. 14), and the lesions disrupt the neurotransmissions from the left hemisphere, which is involved in orolingual motor control. Callosal lesions disrupt the interhemispheric communication that is required for reciprocal inhibition between agonist and antagonist muscles, thus leading to apraxia and uncoordinated oral movements [56].

An association between migraine with aura and AHS was found, and it was attributed to a neuronal depolarization wave and subsequent suppression, which tends to propagate across the cerebral cortex, leading to cortical spreading depression. The CSD wave, which usually involves the posterior parietal cortex, leads to the posterior variant of AHS [57].

**Fig. 15:**
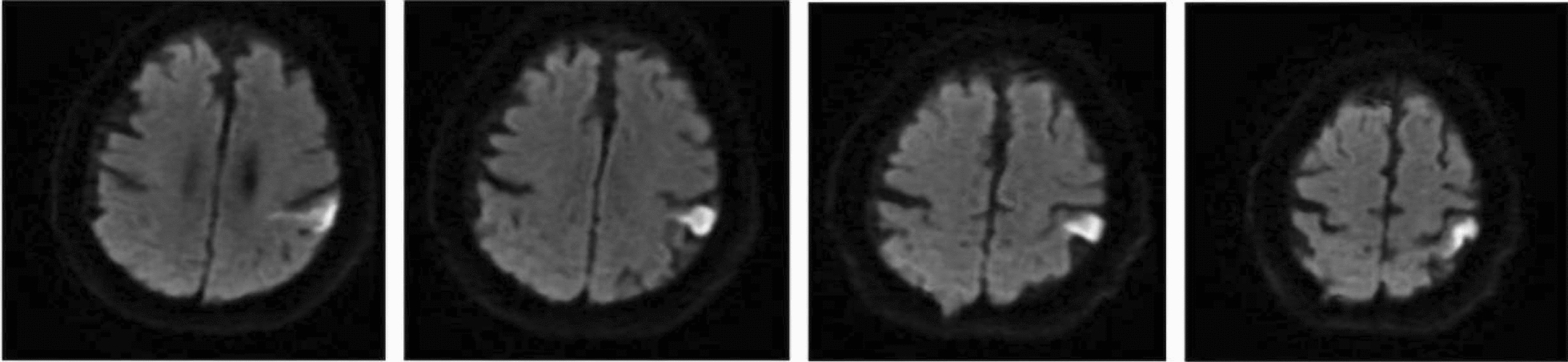
Diffusion-weighted MRI imaging of the patient's brain shows a left-sided parietal stroke [76]

AHS can also manifest with certain atypical clinical manifestations. Higgins et al. (2024) reported a case of a patient who came with symptoms of a levitating hand and an inability to control the voluntary movements of her right hand (Fig. 15) [58].

Alien hand syndrome may be associated with autoimmune pathology. [40] Choker et al. 2014 reported a case where alien hand syndrome was associated with Parry-Romberg syndrome.

Commonly, ischemia of the anterior cerebral artery is associated with the frontal type of alien hand syndrome. However, Alvarez et al. (2020) showed in a case report that ischemia of the anterior cerebral artery can be associated with all three frontal, callosal, and posterior types of alien hand syndrome [59].

Alien hand syndrome can also be associated with chronic alcoholism and Marchiafava-Bignami disease. Shao et al. 2019 presented a case of alien hand syndrome in which a male patient showed uncontrollable forward walking and an inability to stop or turn himself, and gave a history of drinking for 30 years [60].

**Fig. 16:**
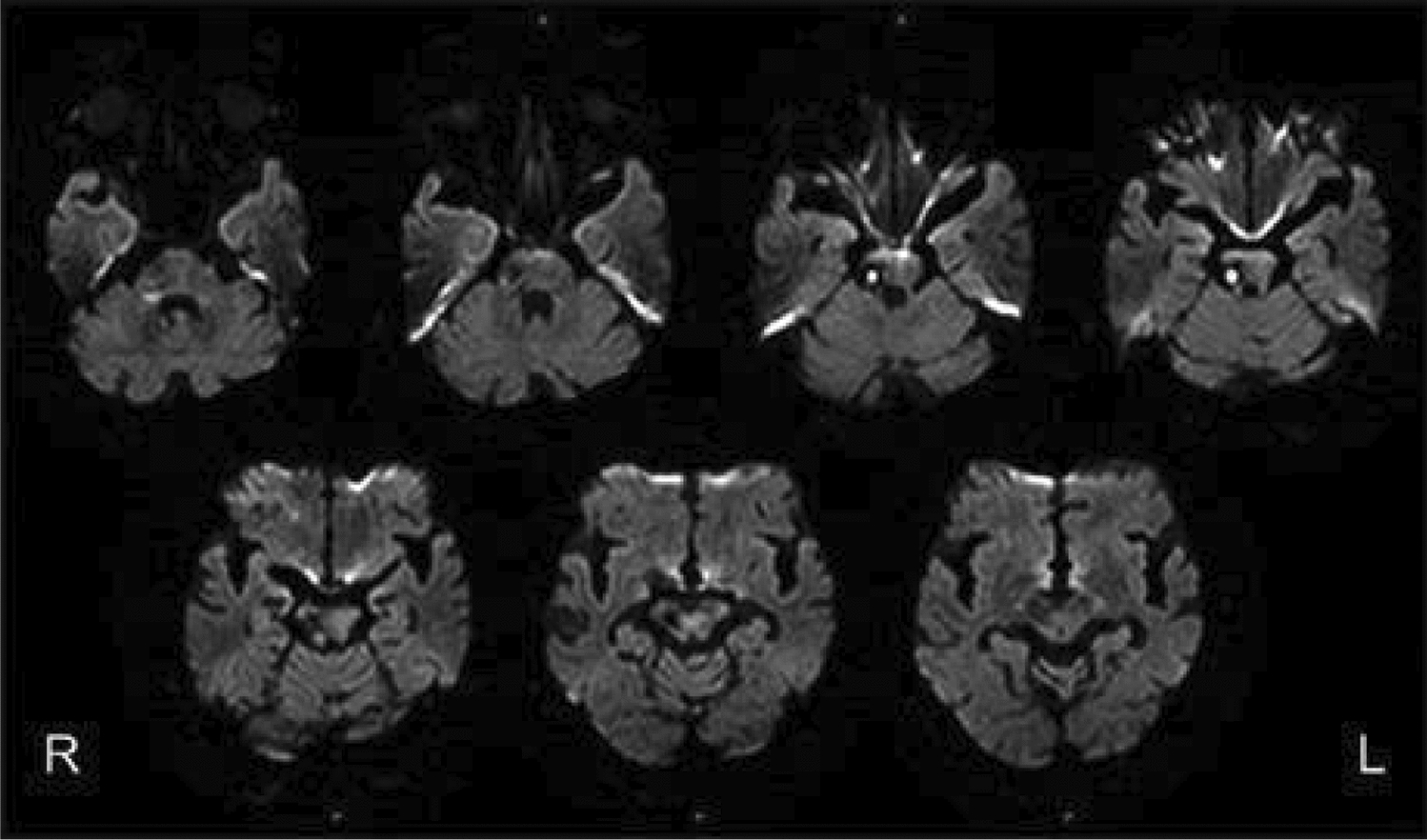
Diffusion-weighted magnetic resonance image showing hemorrhagic foci in the right dorsolateral brain, from the pontine tegmentum to the lower midbrain tegmentum [61]

Hosokawa et al. 2022 showed that pontine hemorrhagic patients may simultaneously develop posterior alien hand syndrome and a feeling of a supernumerary phantom limb. The patient complained of a feeling in the third leg and involuntary movement of the left hand [61] (Fig. 16).

Alien hand syndrome can be associated with corpus callosum syndrome. Faber et al. (2010) presented a case in which a patient developed both corpus callosum syndrome and alien hand syndrome as a result of an infarctive pericallosal lesion [62].

## Relationship between alien hand syndrome and diseases

A comprehensive literature review identified a strong association between Alien Hand Syndrome (AHS) and various cerebral diseases. Vascular events, particularly ischemic strokes, emerged as the most common etiological factors. AHS can be categorized into three variants based on the infarct location: the frontal variant arises from strokes affecting the frontal cortex, the posterior variant from infarctions in the temporoparietal cortex, and the callosal variant from callosal infarctions. Stroke should be considered the primary differential diagnosis when evaluating AHS symptoms, followed by other conditions such as cerebral hemorrhage, aneurysms, and migraines. Neuroimaging plays a pivotal role in identifying the specific brain regions and blood vessels affected by infarction, rupture, or other underlying causes, such as tumors.

In addition to vascular events, AHS has been linked to less common conditions, including rare brain tumors (e.g., oligodendroglioma), neurodegenerative disorders like Creutzfeldt-Jakob disease and sporadic corticobasal degeneration, and rare conditions such as Langerhans cell histiocytosis. Moreover, AHS has been associated with idiopathic conditions like Parry-Romberg syndrome, mood disorders, psychiatric illnesses, diabetes, and cardiac diseases. Orthopedic injuries, which elevate the risk of embolic stroke due to blood clot formation, can also precipitate AHS symptoms in affected individuals.

Our findings underscore the multifactorial nature of AHS, highlighting the necessity of a thorough medical assessment to establish an accurate diagnosis. These diverse etiologies emphasize the importance of a comprehensive diagnostic approach, incorporating clinical evaluation, neuroimaging, and consideration of both common and rare contributing factors.

## Differential diagnosis and etiologies of alien hand syndrome

The symptomatology of Alien Hand Syndrome (AHS) necessitates differentiation from psychiatric disorders, which can pose significant diagnostic challenges [11], particularly when clinicians are unfamiliar with its clinical presentation. AHS may easily be mistaken for several psychiatric conditions, as outlined below:

1. Psychogenic Dystonia: This condition presents with variable symptoms, including pain, weakness, and responsiveness to psychotherapy, suggestion, or placebo. It is often accompanied by multiple somatizations or overt psychiatric disturbances, which can mimic AHS [63].
2. Complete Anesthesia or Deafferentation of a Limb: Patients may deny ownership of the affected limb, manifesting somatic paraphrenia. In these cases, dispossession is often accompanied by delusional beliefs, such as attributing the limb to another person or assigning it a distinct personality. This phenomenon may be mistaken for depersonalization, but differs as the limb’s behavior is perceived to be externally controlled [64].
3. Somatic Delusions and Attentional Disturbances: Somatic delusional disorders, such as monosymptomatic hypochondriacal psychosis, frequently present with complaints of infestation (e.g., parasitosis) or other delusional perceptions, which can overlap with some features of AHS [65].
4. Distortions of Body Schema: Patients may report distortions in the size, shape, or position of a limb, including perceptions of absence, malfunction, or displacement. These disturbances are occasionally observed in psychiatric conditions but differ from the anarchic movements characteristic of AHS [66].
5. Depersonalization-Derealization Disorder: This condition involves emotional detachment or a sense of disconnection from one’s own body, surroundings, or experiences. Patients may describe a sense of unreality or unfamiliarity that superficially resembles the alienation experienced in AHS [67].

For a limb to be classified as “alien” or anarchic in AHS, volitional control must be significantly impaired, accompanied by a sense of external control over the limb’s movements. However, the patient retains their sense of agency over other actions, distinguishing AHS from Schneiderian First-Rank Symptoms, specifically delusions of control/passivity and external control [68]. Other clinical conditions associated with abnormalities in volitional control that must be differentiated from AHS include [68]:

1. Psychogenic Movement Disorders: These disorders involve involuntary movements without an identifiable neuropathological substrate and are generally classified as stereotyped movements [69].
2. Tics: Characterized by involuntary movements or vocalizations that can be partially suppressed with effort, tics differ from the persistent and anarchic movements seen in AHS [69].
3. Choreiform Movements: These consist of brief, transient, and migratory muscle contractions that are not influenced by environmental cues, in contrast to the purposive but uncontrolled actions of the alien limb [69].
4. Xenomelia: This rare condition involves the perception that a limb does not belong to the individual, sometimes accompanied by a desire for amputation. Unlike AHS, volitional control over the limb is preserved [70].
5. Dystonia and Arm Levitation: These movements are typically seen in atypical Parkinsonism syndromes (e.g., progressive supranuclear palsy) and lack the ego-dystonic characteristics of AHS [71].

Imaging studies are crucial in cases where uncommon limb movements are present, particularly when structural lesions such as tumors, stroke, or neurodegenerative processes (e.g., cerebral atrophy) are suspected. AHS has been described as ego-dystonic [70], a psychiatric term denoting thoughts, feelings, or behaviors that are inconsistent with the patient’s sense of self. This contrasts with ego-syntonic symptoms, which are perceived as acceptable and congruent with one’s identity [72].

In severe cases, radical interventions may be necessary to ensure patient safety. For instance, physical restraints have been used in patients with AHS to prevent self-injury or uncontrolled interactions with their surroundings. Furthermore, psychiatric symptoms associated with neurological conditions affecting the brain should also be considered in the differential diagnosis of AHS. Experimental approaches, such as the alien hand experiment or mirror box therapy, may aid in identifying and understanding the unique manifestations of AHS. This thorough differentiation underscores the importance of interdisciplinary collaboration between neurologists and psychiatrists in the accurate diagnosis and management of AHS [72–74] (Table 4).

**Table 4:** Differential diagnosis

Differential diagnosis of alien hand syndrome	
Disorder	Definition/distinguishing features
Category	Manifestations
Delusional disorders/beliefs	Alien limb Loss of identity of a body part Attributing the limb to someone else Separation of a limb Infestation delusions Somatic delusions Schneiderian first-rank symptoms specifically delusions of control/passivity Xenomelia Somatic paraphrenia
Body schema disorders	Limb distortions Shape distortions Displacement/absence of limb Malfunction of the limb Xenomelia (body integrity dysphoria)
Movement disorders	Atypical Parkinsonism Choreiform movements Tics
Other psychiatric disorders	Schizophrenia Depersonalization Psychogenic dystonia

## Conclusion

This review is constrained by its reliance on case reports and small case series, which inherently risk publication bias and lack standardized methodologies. Variability in diagnostic criteria for AHS across studies, particularly in distinguishing sensory vs. motor components, may have influenced case selection. Additionally, heterogeneity in neuroimaging protocols (e.g., MRI vs. CT) and short-term follow-up data limited direct comparisons of lesion localization and long-term outcomes. Despite these limitations, the synthesis of 72 cases clarifies AHS as a disorder rooted in disrupted interhemispheric communication, with three distinct subtypes. Stroke and neurodegeneration are predominant triggers, but rare etiologies (e.g., prion diseases, autoimmune conditions) underscore the need for thorough neuroimaging to exclude mimics like psychogenic movement disorders or somatic delusions. While therapeutic evidence remains anecdotal, this review highlights AHS as a critical “neurological red flag” demanding interdisciplinary collaboration. Future research must prioritize consensus diagnostic criteria, mechanistic studies of cortical disinhibition, and trials of neuromodulation or sensory retraining. By integrating clinical phenomenology with advanced imaging, clinicians can better navigate AHS complexities, improving diagnostic accuracy and paving the way for targeted interventions to restore agency over the alien limb.

## Data Availability

Not applicable, all the data used are within this published article.

## Abbreviations

ACA: Anterior cerebral artery
ADC: Apparent diffusion coefficient
AHS: Alien hand syndrome
CBS: Corticobasal syndrome
CJD: Creutzfeldt-Jakob disease
CSD: Cortical spreading depression
CTA: Computed tomography angiography
CT: Computed tomography
DWI: Diffusion weighted imaging
EEG: Electroencephalogram
FDG: Fluorodeoxyglucose
FLAIR: Fluid attenuated inversion recovery
LBD: Lewy body dementia
MCA: Middle cerebral artery
MRA: Magnetic resonance angiography
MRI: Magnetic resonance imaging
MS: Multiple sclerosis
PCA: Posterior cerebral artery
PCOM: Posterior communicating artery
PMC: Premotor cortex
PRISMA-ScR: Preferred reporting items for systematic reviews and meta-analyses extension for scoping reviews
PSP: Progressive supranuclear palsy
SAH: Subarachnoid hemorrhage
SMA: Supplementary motor area
